# Activities and regulation of peptidoglycan synthases

**DOI:** 10.1098/rstb.2015.0031

**Published:** 2015-10-05

**Authors:** Alexander J. F. Egan, Jacob Biboy, Inge van't Veer, Eefjan Breukink, Waldemar Vollmer

**Affiliations:** 1 Centre for Bacterial Cell Biology, Institute for Cell and Molecular Biosciences, Newcastle University, Richardson Road, Newcastle upon Tyne NE2 4AX, UK; 2 Membrane Biochemistry and Biophysics, Bijvoet Centre for Biomolecular Research, University of Utrecht, Padualaan 8, 3584 Utrecht, The Netherlands

**Keywords:** peptidoglycan, penicillin-binding protein, glycosyltransferase, transpeptidase, carboxypeptidase, lipid II

## Abstract

Peptidoglycan (PG) is an essential component in the cell wall of nearly all bacteria, forming a continuous, mesh-like structure, called the sacculus, around the cytoplasmic membrane to protect the cell from bursting by its turgor. Although PG synthases, the penicillin-binding proteins (PBPs), have been studied for 70 years, useful *in vitro* assays for measuring their activities were established only recently, and these provided the first insights into the regulation of these enzymes. Here, we review the current knowledge on the glycosyltransferase and transpeptidase activities of PG synthases. We provide new data showing that the bifunctional PBP1A and PBP1B from *Escherichia coli* are active upon reconstitution into the membrane environment of proteoliposomes, and that these enzymes also exhibit DD-carboxypeptidase activity in certain conditions. Both novel features are relevant for their functioning within the cell. We also review recent data on the impact of protein–protein interactions and other factors on the activities of PBPs. As an example, we demonstrate a synergistic effect of multiple protein–protein interactions on the glycosyltransferase activity of PBP1B, by its cognate lipoprotein activator LpoB and the essential cell division protein FtsN.

## 1. Introduction

Peptidoglycan (PG) is a key cell wall component in nearly all bacteria, protecting the cell from bursting by its internal turgor and maintaining cell shape [1]. PG consists of glycan strands connected by short peptides and forms a continuous, mesh-like structure around the cytoplasmic membrane, called the sacculus [2]. In Gram-negative species, such as *Escherichia coli*, the sacculus is made of a mainly single layer of PG with a thickness of 3–6 nm, whereas in Gram-positive species, a multi-layered PG is much thicker at 10–20 nm [3]. The glycan strands are made of alternating *N*-acetylglucosamine (Glc*N*Ac) and *N*-acetylmuramic acid (Mur*N*Ac) residues linked by β-1,4 glyosidic bonds. The peptides contain l- and d-amino acids and are linked to Mur*N*Ac residues, the sequence varying across bacterial species. In *E. coli* and most other Gram-negative species, the peptide sequence is as follows: l-Ala-d-iGlu-*m*-Dap-d-Ala-d-Ala (*m*-Dap, *meso*-diaminopimelic acid). Peptides protruding from adjacent glycan strands may be connected, most often from the carboxyl group of d-Ala at position 4 of one peptide to the ε-amino group of the *m*-Dap residue at position 3 of another peptide (3–4 or DD-cross-link).

**Figure 1.**
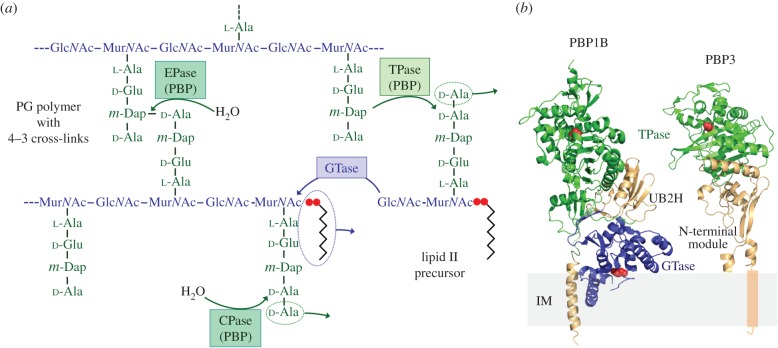
Reactions and domain organization of class A and class B PBPs. (*a*) Peptidoglycan synthesis and peptide cleavage reactions. A nascent glycan strand is synthesized from lipid II precursor by glycosyltransferase (GTase) reactions under the release of the undecaprenol pyrophosphate moiety (indicated by the zigzag line and two red dots). Peptide cross-links are formed by DD-transpeptidase (TPase) reactions catalysed by penicillin-binding proteins (PBPs), forming 4–3 cross-links. Some PBPs are also capable of hydrolysing the terminal d-alanine residue of the pentapeptide stem through DD-carboxypeptidase (CPase) activity, or hydrolysing the 4–3 cross-link through DD-endopeptidase (EPase) activity. (*b*) Crystal structures of *E. coli* PBP1B and PBP3. The bifunctional PBP1B (PDB ID: 3FWM) and the TPase PBP3 (PDB ID: 4BJP) anchor to the inner membrane (IM). The GTase domain of PBP1B is shown in blue, the TPase domains of both proteins are shown in green. The non-catalytic/regulatory domains such as the UB2H domain of PBP1B or the N-terminal module of PBP3 are shown in wheat. The residues essential for catalytic activity in each domain are labelled in red.

During cell growth and division, the surface of the sacculus is enlarged by the incorporation of new PG material. In this process, the precursor lipid II (undecaprenyl-pyrophosphoryl-Mur*N*Ac(pentapeptide)-Glc*N*Ac) is polymerized by glycosyltransferase (GTase) reactions, under the release of undecaprenol pyrophosphate, and peptide cross-links are formed by transpeptidase (TPase) reactions (figure 1*a*). TPase involves a donor pentapeptide, which loses its carboxy-terminal d-alanine residue in the course of the reaction, and an acceptor peptide with a free amino group [4]. TPase reactions connect peptides between newly polymerized glycan strands, but also between peptides in new strands and old ones in the sacculus; the latter attaches the new PG to the sacculus. PG hydrolases are required to open the PG net allowing the insertion of the newly attached PG into the stress-bearing layer and sacculus growth. Presumably, PG growth is facilitated by dynamic multi-enzyme complexes that contain all the enzymatic activities required, and that are tightly regulated and coordinated with cell growth [5,6].

PG synthases have a modular structure and are classified according to their activity [4,7]. Class A penicillin-binding proteins (PBPs) are bifunctional enzymes with both GTase and TPase activity, whereas class B PBPs and Mgt enzymes are monofunctional TPases and GTases, respectively. Class A and class B PBPs are anchored to the cytoplasmic membrane by a single transmembrane region near their N-terminus. Most of them also have non-catalytic domains of which some are involved in interactions with other proteins to regulate the enzymatic activities (figure 1*b*) [8]. The abundant PG hydrolases come with different specificities for cleavage in PG and have a number of cellular roles [9]. Class C PBPs are PG hydrolases with DD-carboxypeptidase (CPase) or endopeptidase (EPase) activity (figure 1*a*). EPases cleave the peptide cross-links, and CPases trim the peptides in the PG by hydrolysing the terminal amino acid residue.

Here, we provide a brief overview of the pioneering work in the past century on the activities of PG synthases. We then review the current knowledge on the activities of these enzymes studied by *in vitro* PG synthesis assays. This work was only possible after optimizing the isolation of the enzymes and the substrate, lipid II. We also present previously unpublished data demonstrating that PBP1A and PBP1B from *E. coli* are active when reconstituted into proteoliposomes, and that both enzymes exhibit DD-CPase activity under certain conditions. Another main focus is how multiple interactions with other proteins regulate the activities of PBPs, to ensure coordination of PG growth with cell growth and other cellular processes, such as outer membrane (OM) constriction. In line with this, we also present previously unpublished data demonstrating a synergistic stimulatory effect of two binding partners on the GTase activity of PBP1B.

## 2. Early work on the activities of peptidoglycan synthases

Here, we provide a brief overview of the early work on the activities of PG synthases, which is not complete and not always in a chronological order. Historically, the work on PG synthases began after it became clear that penicillin, the powerful antibiotic discovered by Alexander Fleming in 1929 [10], is a specific inhibitor of bacterial cell wall synthesis [11–13]. With the knowledge of the chemical structure of the PG precursors [14] and the basic subunits present within the high-molecular PG [15], it became clear that the final step in PG synthesis requires two enzymatic activities, glycosyltransferase (GTase, or transglycosylation) reactions for glycan strand polymerization and transpeptidation (TPase) for peptide cross-linking. Further early studies revealed that penicillin treatment resulted in the inhibition of TPase resulting in the formation of uncross-linked PG [16–18]. To the best of our knowledge, *in vitro* PG synthesis reactions were first demonstrated in the year 1966 when Izaki *et al*. [19] used radiolabelled UDP-Mur*N*Ac-pentapeptide as substrate for a crude enzyme preparation (possibly a membrane extract) from *E. coli* and obtained high-molecular weight products under release of d-Ala. This and further studies from the Strominger laboratory with membrane fractions from *E. coli* helped to elucidate the mechanism of the TPase reaction and the mode of action of penicillin.

Membrane preparations from other bacteria were also investigated. Initial work showed that membrane fractions from *Staphylococcus aureus* and *Micrococcus luteus* produced linear, uncross-linked glycan strands [20,21], but subsequent studies using a cell wall–membrane complex from these species demonstrated both GTase and TPase activities [22,23]. This suggested that in these reactions the cross-linking occurred mainly between peptides of the newly made PG and peptides in the pre-existing cell wall. Other work provided evidence for PG synthesis activity and its inhibition by various antibiotics in membranes or membrane extracts from *Gaffkya homari* [24,25], *Bacillus megaterium* [26] and *E. coli* [27–30]. Cell membranes from *E. coli* were also used to assay the TPase activity of PBP2, which is selectively inhibited by the β-lactam mecillinam [31].

PG synthesis reactions can also be performed in cells of *E. coli* or other bacteria that were treated with ether, which increases the permeability of the cytoplasmic membrane permitting the access of extracellular, radiolabelled precursors to other cellular precursors and enzymes [32]. The radiolabelled compound can be *meso*-diaminopimelic acid (*m*-Dap) or UDP-Mur*N*Ac-pentapeptide, and the detection of the product occurs by hydrolysis with a muramidase followed by paper or high-pressure liquid chromatography. The technique showed the synthesis of PG in ether-treated cells of *Proteus mirabilis* [33] and *E. coli* [34–36]. In the latter, two types of TPase reactions were observed, presumably by enzymes that differed in their sensitivity to penicillin G and capability to form hypercross-linked PG versus cross-links between two peptides only (to form peptide dimers) [35].

The study of *in vitro* activities of DD-transpeptidases can be cumbersome as most of these enzymes are membrane-anchored and difficult to purify in sufficient quantity for biochemical work, and they require ongoing glycosyltransferase activity and/or complex substrates (see below). A set of enzymes from *Streptomyces* strains became early models to study transpeptidation because they were water-soluble and used small, soluble peptides as substrates. These were the DD-carboxy-/transpeptidases from the *Streptomyces* strains R61, K11 and R39. These enzymes can use the small donor diacetyl-l-lysine-d-alanine-d-alanine (with radioactive label at the acetyl groups) for transpeptidation reactions with a variety of possible acceptors, which could be glycine, d-(but not l-) amino acids such as d-alanine or *m*-Dap, or di- or oligo-peptides containing glycine or d-alanine [37]. Further work characterized the kinetics of the TPase and hydrolase (carboxypeptidase, CPase) activities, and determined the specificity of the TPase enzymes regarding the acceptor peptides that resembled structures found in the PG [38–40].

The lipid intermediates in PG synthesis (lipid I and lipid II) were identified by the mid-1960s [41,42], but possibly owing to their limited availability and the lack of purified enzymes it took some time until lipid II was used as substrate for PG synthesis reactions. The laboratory of Michio Matsuashi pioneered the semi-purification of peptidoglycan synthases (PBPs) via binding to ampicillin–sepharose, followed by elution of the proteins with hydroxylamine and activity assays with lipid II substrate [43]. The selectivity for certain synthases was achieved by using mutant strains overexpressing or depleting some of the PBPs. This impressive work led to the first characterization of the bi-functional PBPs from *E. coli*, PBP1A [44] and PBP1B [43], showing the time-course of lipid II consumption and formation of PG, as well as the antibiotic inhibition of GTase and TPase by measuring the extent of cross-linkage in the product formed [45,46]. This approach was also undertaken to measure the activity of PBP3 from *E. coli*, a monofunctional transpeptidase. However, this work reported GTase and TPase activity for PBP3 [47], suggesting that the PBP3 preparation contained a contaminating GTase activity, presumably the bifunctional PBP1B which is now known to interact with PBP3 [48]. Consistent with this, a second study with semi-purified PBPs detected PG synthesis activity for PBP1A and PBP1B, but not for PBP3 alone [49]. Using lipid II as substrate, various purified enzymes from Gram-positive *Bacillus* species were also assayed. These produced uncross-linked or poorly cross-linked PG [50,51]. Presumably, the TPase efficiency was low in these experiments, because, as we know now, some enzymes from Gram-positive species require amidated lipid II substrate (see below) which was not available in the 1980s.

PG biosynthesis is a validated target for antibiotics. The highly successful class of β-lactam antibiotics inhibits the TPase, but there are only few known inhibitors of the essential GTase reactions, for example the lipid II analogue moenomycin (flavomycin). Several ‘crude’ assays were established to screen for GTase inhibitors, using cell membrane (extract) without purifying the enzymes. One of these assays uses cell extract from a MurG overexpressing *E. coli* strain and UDP-Mur*N*Ac pentapeptide from *Enterococcus faecium*, which harbours an l-lysine residue at position 3 and is therefore not a substrate for the *E. coli* TPase [52]. The addition of radiolabelled UDP-Glc*N*Ac initiates the synthesis of lipid II which is used by the *E. coli* GTases to produce uncross-linked glycan strands; these can be quantified by paper chromatography where they remain at the start spot. Another, rather simple assay for GTase inhibitors is based on a competition with moenomycin for binding to the active site [53]. For this assay, the bifunctional PBP1A and PBP1B present in crude *E. coli* membrane extract (of wild-type or mutant strains) are labelled with a radioactive or fluorescent β-lactam. This extract is incubated with beads containing coupled moenomycin in the wells of a filter microplate, followed by washing and quantification of the bound PBPs by scintillation counting or fluorescence measurements. Compounds binding to the GTase active site compete with moenomycin thereby reducing the signal.

In the following sections, we provide an overview on the methods to isolate lipid II, the currently used *in vitro* PG synthesis assays using lipid II or its derivatives as substrate, and the major findings on the GTase and TPase reactions obtained with these assays.

## 3. Isolation of lipid II for *in vitro* assays

### (a) Unlabelled lipid II

**Figure 2.**
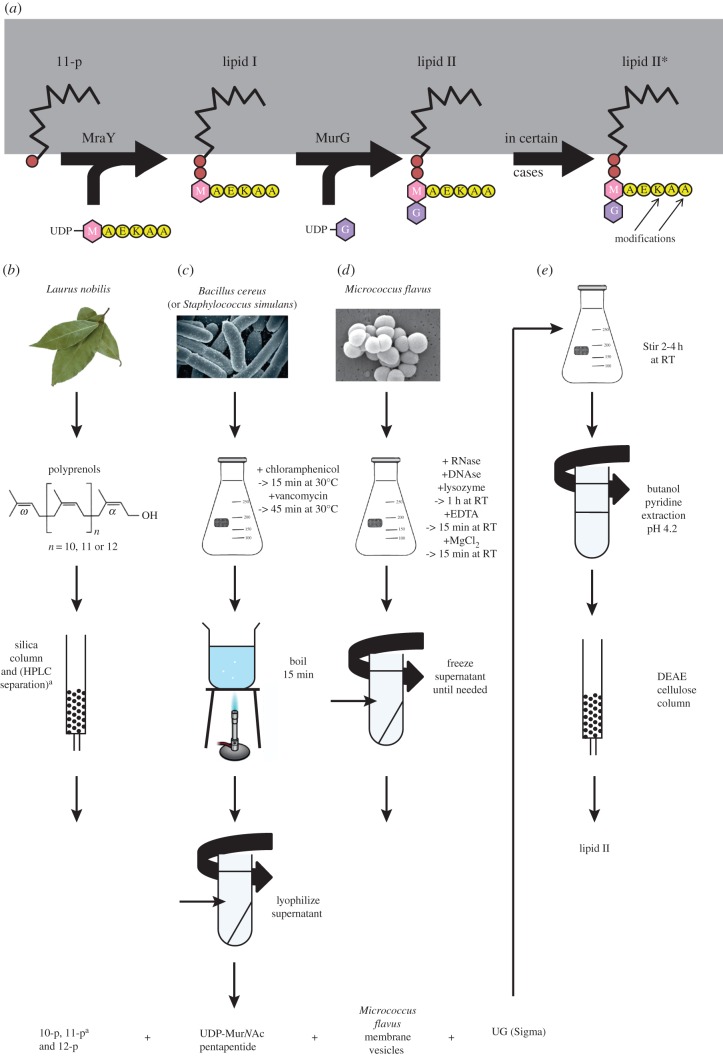
Synthesis of lipid II. (*a*) Schematic of the membrane-associated steps of lipid II synthesis. UDP-Mur*N*Ac-pentapeptide is attached to the lipid carrier undecaprenol phosphate by MraY creating lipid I. Next, UDP-Glc*N*Ac is attached to lipid I by MurG creating lipid II, which can be modified at different positions as described in the text. (*b*) Undecaprenol can be extracted from leaves of *Laurus nobilis* using a mixture of acetone and hexane, followed by purification over a silica column and, if a uniform chain length is needed, by reversed-phase high-pressure liquid chromatography (HPLC). (*c*) UDP-Mur*N*Ac-pentapeptide can be extracted from *Bacillus cereus* (for the *m*-Dap version) or *Staphylococcus simulans* (l-lysine version) by blocking cell wall synthesis with vancomycin and subsequent boiling of the cells in water, centrifugation and lyophilization of the UDP-Mur*N*Ac-pentapeptide supernatant. (*d*) MraY and MurG are present in membranes from *Micrococcus flavus*, which are lysed and centrifuged to obtain the membrane vesicles in the supernatant. (*e*) To produce lipid II, all components are incubated for 2–4 h at room temperature, lipids are extracted using butanol/pyridine at pH 4.2, and lipid II is purified over a DEAE cellulose column.

The partial or total chemical synthesis of lipid II has been described [54–56]. However, the biochemical production of lipid II is easier and more feasible for non-chemists. Therefore, we focus in this review on the available biochemical methods of obtaining (labelled) lipid II (figure 2).

The biochemical production of lipid II requires four components, the lipid carrier undecaprenyl phosphate (11-p), UDP-Mur*N*Ac-pentapeptide, UDP-Glc*N*Ac and the enzymes catalysing the formation of lipid II from these substrates, MraY and MurG (figure 2*a*). For a long time, the bottleneck in the (bio)chemical production of lipid II has been the availability of the lipid tail of lipid II, which is a phosphorylated C_55_ isoprenoid alcohol (made of 11 prenoid units), also called undecaprenyl phosphate or bactoprenol, in both Gram-positive and -negative bacteria [42,57]. The early protocols for the biochemical lipid II synthesis used membrane vesicles from Gram-positive bacteria as the source of this lipid, and these vesicles also provided the MraY and MurG [58,59]. However, although membranes from Gram-positive bacteria contain more undecaprenyl phosphate than those from Gram-negatives [60], the cellular pool is small because it is constantly being recycled during the cell wall synthesis cycle, limiting the amount bacteria need for growth [61,62]. As a result, to obtain approximately 150 µg undecaprenyl phosphate, large amounts of membranes and reaction/extraction volumes (1 l) and large anion exchange column sizes (2.5 × 25 cm) were needed for the production, extraction and purification of lipid II [59]. The yield of lipid II in the biochemical synthesis could be considerably improved (by at least a factor of 100) by the addition of purified undecaprenyl phosphate [63]. Moreover, the substrate specificity of MraY for polyisoprenoid phosphates turned out to be so broad that lipid II variants could be prepared with polyisoprene tails that vary from two to more than 20 isoprene units [63].

Plant leaves are the best source for polyisoprenoids [64], and there are two easily accessible sources for undecaprenol: bay leaves and leaves from the Staghorn sumac (*Rhus typhia*), an indigenous plant in North America and ornamental in Europe. The extraction and purification of undecaprenol from plant leaves is relatively straightforward [65,66]. Ground leaves are extracted with a mixture of acetone and hexane followed by silica column purification. The prenols are present in a mixture of polyisoprenoids of 10, 11 and 12 isoprene units. A uniform prenoid length can be obtained in a second purification step using reversed-phase high-pressure liquid chromatography (HPLC; figure 2*b*). Subsequent phosphorylation to the polyprenol phosphate can be performed in a single step [67].

UDP-Glc*N*Ac can be purchased from different chemical supply companies. UDP-Mur*N*Ac pentapeptide is extracted from bacteria and its source is dependent on the chemical version of lipid II that is desired. *Bacillus cereus* and *Staphylococcus simulans* are the best sources for the extraction of the lipid II versions with *m*-Dap and l-lysine, respectively [1]. The extraction of UDP-Mur*N*Ac pentapeptide (figure 2*c*) is based on its cellular accumulation upon inhibition of cell wall synthesis by vancomycin [68]. UDP-Mur*N*Ac pentapeptide is extracted by boiling the cells in water followed by centrifugation and lyophilization of the supernatant. This UDP-Mur*N*Ac pentapeptide-containing extract can directly be used for the synthesis of lipid II without further purification.

The enzymes MraY and MurG can come from various sources ranging from the purified enzymes to isolated bacterial membranes from Gram-positive bacteria or *E. coli* cells overexpressing the two proteins [69]. Membranes from Gram-positive bacteria are easiest to work with, and most often *Micrococcus flavus* membranes are used (figure 2*d*). However, some membrane preparations lack sufficient MurG activity; this is the case for membranes from *Bacillus subtilis* which only synthesize lipid I (E. Breukink, unpublished data).

To finally produce lipid II, the four components mentioned above are mixed in a detergent containing buffer and stirred for 2–4 h at room temperature (figure 2*e*). Lipid II is extracted with butanol/pyridine at pH 4.2 according to the procedure originally developed by Strominger and co-workers [58] and is further purified in one step using a small anion exchange column [63].

### (b) Labelled lipid II

In order to track lipid II or the products formed by PG synthases in functional assays (see below), fluorescent groups or radioactive isotopes can be incorporated in the UDP-Mur*N*Ac pentapeptide and/or UDP-Glc*N*Ac moieties. Fluorescent labelling of lipid II can be achieved by the incorporation of fluorescently labelled UDP-Mur*N*Ac pentapeptide which, for example, contains a pyrene [63], a dansyl [70] or a 7-nitro-2,1,3-benzoxadiazol-4-yl label [71]. This approach requires purification of the l-lysine version of UDP-Mur*N*Ac pentapeptide which can be labelled at the ε-amino group of l-Lys followed by purification of the labelled precursor [63,70]. However, this approach is rather inefficient as the labelled UDP-Mur*N*Ac pentapeptide is used in excess during the synthesis of labelled lipid II. Novel approaches using the biorthogonal click chemistry significantly increase the efficiency of labelling. For this, the amino group of the lysine residue of lipid II was converted into an azide via the incubation with imidazole-1-sulfonyl azide hydrochloride, which acts as a diazo donor in the conversion of primary amines into azides [72]. The azido-lipid II is extracted and purified from the reaction mixture, and can then be used in (copper catalysed) click reactions with azide or cyclooctyne containing fluorophore labels.

Radiolabelled UDP-Glc*N*Ac can also be incorporated into lipid II [70]. While producing radiolabelled lipid II using [^14^C]-Glc*N*Ac, it was observed that MurG is able to exchange the Glc*N*Ac of the lipid II head group with Glc*N*Ac of UDP-Glc*N*Ac. This property has been used to obtain lipid II with a radiolabelled Glc*N*Ac by a simple exchange reaction using purified lipid II and [^14^C]-Glc*N*Ac in the presence of MurG (E. Breukink, unpublished data).

### (c) Biological variants of lipid II

Next to the variation in the amino acid composition of the pentapeptide, some bacteria modify one or more of the carboxylic groups of the stem peptide by amidation [1]. In *S. aureus*, the MurT/GatD complex amidates the d-Glu residue [73,74], and in *B. subtilis* AsnB amidates the carboxylic group of *m*-Dap (van Bentum and Breukink, unpublished data). Lipid II versions carrying these amidations can be generated *in vitro* by incubating lipid II in the presence of amidating enzymes, ATP and an amido group donor such as glutamine or even ammonia [75].

Peptide branches linked to position 3 of the stem peptide are another example of species-specific modification of lipid II. These branches are made of one to seven amino acids; a well-known version is the pentaglycine peptide present in the PG of *S. aureus*. In this species, the peptide branch is attached to lipid II by the successive action of the Fem transferases, FemX, FemA and FemB, that use amino acid loaded t-RNAs as substrates [76]. FemX attaches the first glycine residue to lipid II, the second and third residue are attached by FemA, which recognizes only lipid II with a previously attached first glycine residue. The last two glycine residues are attached by FemB, which also has acceptor specificity and only uses lipid II with three glycine residues attached. The attachment of the branch to lipid II has been achieved *in vitro* using purified lipid II and enzymes [77]. A similar system has been shown to exist for the bacterium *Weissella viridescens*, in which Fem enzymes use UDP-Mur*N*Ac pentapeptide as the substrate for the attachment of the first alanine followed by the attachment of a serine and alanine at the level of lipid II by unknown Fem-like proteins. The first step of this process has been reconstituted *in vitro* [78].

The synthesis of significant amounts of native, unmodified or modified lipid II or of versions carrying reporter groups has provided essential tools for studying the PG synthesis pathway and generated crucial knowledge about the enzymes involved and their regulation. Examples for the successful use of lipid II versions are outlined below.

## 4. Processivity and substrate specificity of GTases

**Figure 3.**
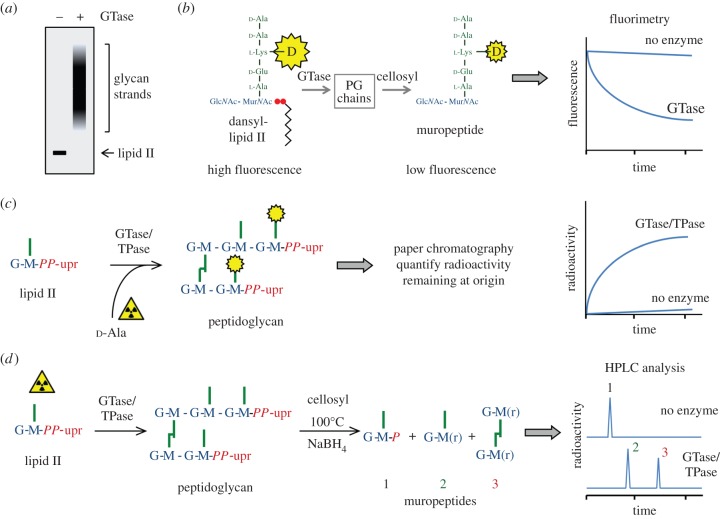
Schemes of the currently used PG synthesis activity assays. (*a*) Separation of glycan strands by SDS–PAGE. (*b*) Continuous GTase assay with dansylated lipid II substrate. (*c*) Exchange reaction with radiolabelled d-Ala to monitor TPase activity. (*d*) *In vitro* GTase/TPase assay using lipid II substrate, with muramidase digestion and analysis of the resulting muropeptides by HPLC. Alternatively, the PG produced can be quantified by paper chromatography as shown in (*c*).

Figure 3 summarizes the currently often used *in vitro* PG synthesis assays. PG GTases belong to the glycosyltransferase family 51 (GT51) [79] and polymerize lipid II to produce glycan strands that, in the absence of TPase activity, contain an uncross-linked pentapeptide at each Mur*N*Ac residue. When testing bifunctional GTase/TPases, uncross-linked glycan strands can be obtained by the addition of a β-lactam antibiotic. Lipid II and glycan strands containing two to approximately 20 disaccharide units can be separated by sodium dodecyl sulfate polyacrylamide gel electrophoresis (SDS–PAGE) [80] (figure 3*a*). Higher oligomers are usually not separated. If the lipid II substrate is radiolabelled, the products of the reaction separated by SDS–PAGE can be detected by autoradiography of the dried gel, and the bands can be quantified by densitometric analysis.

The glycan strand grows by the addition of the next subunit to the Mur*N*Ac end of the growing strand, whereby the growing glycan strand serves as donor and the next lipid II as acceptor in the reaction [81–83]. This is consistent with crystal structures showing the donor and acceptor binding sites [79,84]. Analysis of the time-course of GTase reactions suggest that the enzymes work processively, i.e. the growing glycan strand remains at the enzyme active site until it is fully polymerized and released. When starting with lipid II, the initiation of the reaction requires the simultaneous binding of two lipid II molecules to the acceptor and donor sites of the GTase. Following the first GTase reaction and upon release of the undecaprenol pyrophosphate, the tetrasaccharide product moves into the enzyme's donor site allowing the binding of the next lipid II into the acceptor site and the next GTase reaction to proceed, yielding the hexasaccharide. These processive GTase reactions are faster than the initiation of the reaction with two lipid II molecules. Tetrasaccharide- and hexasaccharide-containing precursors (lipid IV or lipid VI) are poor or no substrates on their own, but can be incorporated into higher oligomers when lipid II is polymerized [80,85]. It has been recently shown for the monofunctional *S. aureus* GTase MtgA that a low concentration of lipid II (binding to the acceptor site) increases the binding affinity of moenomycin to the donor site, indicating an allosteric activation of the donor site and positive cooperativity between both sites [86].

Different GTases differ in their processivity resulting in glycan strands with different length distribution. PBP1A from *Aquifex aeolicus* produces long glycan strands as does PBP1A from *E. coli*, but the distribution of the latter is narrower. A series of *A. aeolicus* PBP1A single point mutants differed significantly in the extent of lipid II conversion and product length distribution and allowed the identification of amino acid residues crucial for GTase activity [80]. The bifunctional GTase/TPase PBP2 from *S. aureus* was found to be mutated after selection for increased sensitivity to moenomycin, a GTase inhibitor. One of these PBP2 mutants (Y196D) produced shorter glycan strands *in vitro*. In the cell, the defective GTase of PBP2(Y196D) needs to be compensated by the presence and activity of the monofunctional GTase SgtB. Hence, presumably the two PG synthases cooperate in the cell, and this cooperation is essential in the presence of the mutated PBP2 to produce glycan strands of appropriate length and cross-link these through PBP2's TPase activity [87].

## 5. Kinetic characterization of GTases

Using radioactive- or fluorescence-labelled lipid II permitted the kinetic characterization of some GTases. PBP2 from *S. aureus* was most active at pH 5.0 and with radiolabelled lipid II displayed a *K*_m_ of 4 µM, *k*_cat_ of 0.015 s^−1^ and *k*_cat_/*K*_m_ of 3400 M^−1^ s^−1^ [88]. The *S. aureus* monofunctional GTase Mgt (also termed MtgA) becomes essential in the absence of PBP2 and has similar kinetic properties to PBP2 [89,90]. Compared with these enzymes, the bifunctional GTase/TPases PBP4 from *Listeria monocytogenes* and PBP1B from *E. coli* had either lower or higher efficiency (PBP4: 1400 M^−1^ s^−1^; PBP1B: 39 000 M^−1^ s^−1^) [91,92].

A major breakthrough in GTase assays came from the development of a continuous assay using fluorescent dansyl-lipid II (the dansyl group resides at the ε-amino group of lysine at position 3 of the peptide) [93]. In this assay, polymerization of dansyl-lipid II to glycan strands followed by their digestion with a muramidase (such as mutanolysin or cellosyl) results in the formation of dansylated muropeptide that shows a lower fluorescence than the lipid II substrate owing to the reduced fluorescence intensity of the dansyl group upon removal of the lipid moiety (figure 3*b*). Hence, the progression of the GTase reaction can be followed by the reduction in fluorescence over time. The assay was first used to characterize PBP1B, demonstrating its pH optimum at pH 8.5–9.5, the effect of detergents, DMSO and divalent cations on GTase rate, and determining the catalytic constants. However, the caveat with this study is that the source of PBP1B is not clear. While the title and results section designates it as *E. coli* PBP1B, the methods section reports that it was the PBP1b gene from *Streptococcus pneumoniae* which was overexpressed and purified from *E. coli* [54,93]. So far, we have been unable to obtain information from the authors to clarify the nature of the enzyme used in these studies. The continuous GTase assay was later modified for use with a microplate reader for higher sample throughput [94]. This allowed a screen for the optimal detergent and DMSO concentrations for PBP1a from *Thermotoga maritima*, which was also shown to produce significantly shorter glycan strands than *E. coli* PBP1B using the SDS–PAGE system to analyse the products of radiolabelled lipid II [94]. Purified PBP2a from *S. pneumoniae* was active with fluorescent dansyl-lipid II with some but not all tested detergents, and the products could be analysed by SDS–PAGE (replacing the use of radiolabelled lipid II) [95]. Interestingly, this work also showed that a truncated version of PBP2a lacking the membrane anchor was severely affected in GTase activity, producing shorter glycan strands, indicating that the transmembrane region is crucial for GTase activity. This continuous assay is particularly useful to evaluate the relative effect of interaction partners on the activity of *E. coli* GTases which will be described below.

## 6. Measurement of TPase activity

TPase reactions lead to the formation of peptide cross-links under the release of d-alanine and are catalysed by class A and class B PBPs. The reaction requires a pentapeptide donor that loses the terminal d-alanine residue resulting in an intermediary tetrapeptide bound to the catalytic serine of the PBP. Nucleophilic attack of an amino group of the acceptor peptide resolves the intermediate and generates the new peptide bond. The acceptor can, in principle, be a tri-, tetra- or pentapeptide with or without a branch at position 3, and can be either a monomer or an already cross-linked peptide. In the latter case, multimeric peptide cross-links (such as trimers, tetramers, etc.) are produced. Depending on the reaction conditions, some TPases also accept a water molecule as acceptor, acting as a carboxypeptidase (CPase) to trim the pentapeptide donor to a tetrapeptide (see below). Hence, the release of d-alanine alone cannot distinguish between TPase and CPase activity, and the cross-linked transpeptidation product needs to be detected to unambiguously prove TPase activity.

Apart from lipid II, TPases can use other substrates such as peptide or thiolester donors, and d-amino acid acceptors, which are mainly used for kinetic characterization. In these TPase exchange reactions, the donor (benzoyl-Gly-thiolactate or benzoyl-Gly-glycolate) or a peptide with a d-alanyl-d-alanine terminus reacts with a d-amino acid acceptor (often d-alanine) followed by quantification of the products [27,91,96]. GTase/TPases incorporated d-amino acids even in the presence of lipid II, i.e. they perform transpeptidation reactions with d-amino acids under the release of d-alanine, incorporating the other d-amino acid. If the added amino acid is radiolabelled d-alanine and lipid II is not, radioactivity becomes incorporated into the pentapeptides of the newly made PG (figure 3*c*). Hence, the incorporation of radiolabelled d-alanine or d-tryptophan has been used to detect or quantify TPase activity [97–99]. Interestingly, fluorescence labelled d-amino acids can be incorporated into the PG of live bacteria during growth allowing the visualization of the incorporation sites and determination of the modes of PG growth in a variety of bacterial species [100–102].

 In a bacterial cell, GTases and TPases use lipid II to synthesize a high-molecular weight PG product. This reaction has been reconstituted *in vitro*, followed by the analysis of the composition of the PG synthesized [103,104]. For this, the reaction of a PG synthase with radiolabelled lipid II is stopped by boiling at mild acidic pH, which also hydrolyses the pyrophosphate moiety of the lipid anchor (and of unused lipid II), leaving one phosphate residue at the reducing end of the glycan strands. Incubation with a muramidase (cellosyl or mutanolysin) releases the muropeptides, which are reduced with sodium borohydride to change Mur*N*Ac to *N*-acetylmuramitol, followed by HPLC analysis using a radioactivity flow-through detector (figure 3*d*). Quantification of the separated muropeptide peaks allows the calculation of the average length of the glycan strands, the extent of peptide cross-linkage and whether higher oligomers such as trimers or tetramers are formed [103,104]. The assay also detects possible CPase products, and allows the measurement of the effect of interacting proteins on TPase activity (see below).

## 7. GTase and TPase activities of class A penicillin-binding proteins

Class A PBPs exhibit both GTase and TPase activities, which is readily observed when such an enzyme polymerizes a mixture of dansyl-lipid II and an excess of unlabelled lipid II, producing a high-molecular weight fluorescent PG product that barely enters the SDS–PAGE [95]. This product contains polymerized glycan strands and peptide cross-links, and is not obtained in samples containing a β-lactam blocking TPase. However, this technique cannot quantify the extent of cross-linking in the PG produced.

Both of the PG synthesis activities of class A PBPs have been demonstrated with their natural substrate *in vitro*. PBP1A and PBP1B of *E. coli* were capable of polymerizing lipid II to form PG by the GTase–TPase assay with radiolabelled lipid II (figure 3*d*) [103,104]. For PBP1A, the PG product contained glycan strands approximately 20 disaccharide units in length with approximately 18–26% of peptides present in cross-links [104]. PBP1B was shown to be more active at conditions which favour dimerization, and produced a PG product with glycan strands more than 25 disaccharide units in length and with approximately 50% of the peptides present in cross-links [103]. Although these enzymes are semi-redundant in the cell at standard laboratory conditions, there are differences in their activities. In a time-course experiment studying PBP1A activity, only GTase was observed within the first 15 min of the reaction, with the consumption of approximately 25% of the available substrate [104]. Significant TPase activity was observed only after this initial period of glycan strand production, suggesting that PBP1A requires pre-oligomerized or high-molecular weight PG as acceptor for TPase reactions. Consistent with this observation, PBP1A was able to attach approximately 25% of the newly synthesized material to existing PG sacculi added to the reaction [104]. Attachment of the new material occurred by TPase reactions, with monomeric tri- and tetrapeptides in the sacculi acting as acceptors and the pentapeptides of the newly made glycan strands acting as donors. In contrast, PBP1B produced cross-linked material from the onset of the reaction [103]. This difference may, in part, be due to the dimerization of PBP1B, which was not observed for PBP1A at the reaction conditions. A dimer of PBP1B could potentially synthesize two glycan strands, which could be simultaneously linked together by TPase reactions. The coupling between GTase and TPase reactions is supported by the crystal structure of PBP1B in complex with the GTase inhibitor moenomycin, which occupies the GTase donor site as does the nascent glycan strand. Its orientation suggests that the growing glycan strand is produced such that peptides are brought within range of the TPase active site [105].

Both PBP1A and PBP1B are able to catalyse the polymerization of lipid II into glycan strands in the absence of TPase activity, with the active site either blocked by β-lactam (e.g. penicillin) or inactivated by mutation of the key catalytic serine residue (PBP1A, S473; PBP1B, S510) [91,103,104]. In contrast, no or significantly reduced TPase activity is observed when the GTase activity is blocked by inhibition with moenomycin or by mutation of the key catalytic glutamate (PBP1A, E94; PBP1B, E233), although the enzyme still binds β-lactam antibiotic indicating a properly folded TPase site. Moreover, the native enzyme does not significantly cross-link already polymerized glycan strands (with monomeric peptides), and mixing an inactive TPase version of PBP1B (capable of synthesizing uncross-linked glycan strands) with GTase mutant (with functional TPase site) does not result in significant TPase activity. These data show that TPase activity of the bifunctional PG synthases requires ongoing GTase activity in the same molecule of the enzyme [91,103,104] and is consistent with the PBP1B structure-based model that the growing glycan strand produced by the GTase site of the enzyme delivers its peptides in its TPase site for peptide cross-linking [105].

## 8. Class A penicillin-binding proteins exhibit carboxypeptidase activity

We have previously observed a low percentage of CPase products (muropeptides with monomeric tetrapeptides and dimeric tetratetrapeptides, respectively) in the reaction products of *E. coli* PBP1A with lipid II, and CPase products were enhanced in the presence of its regulator LpoA (see section ‘Regulation of PBP activity’) [106]. Such an activity is possible considering the similarity of the TPase domain of class A PBPs with the CPase domain of class C PBPs, and that the first step of both reactions involves the binding of the same pentapeptide donor to the active site serine residue, under release of the terminal d-alanine residue. TPases transfer the enzyme-bound tetrapeptide to a peptide acceptor, and CPases transfer the tetrapeptide to a water molecule releasing the tetrapeptide from the enzyme.

**Figure 4.**
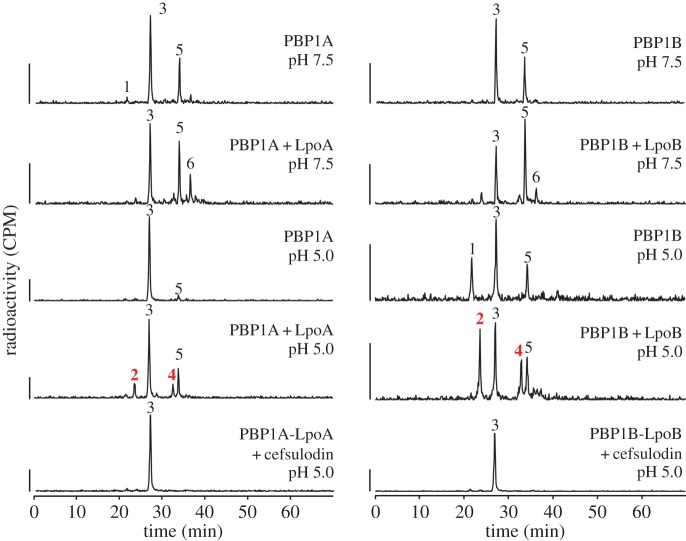
Class A PBPs exhibit carboxypeptidase activity at pH 5.0. Representative examples of HPLC chromatograms from PG synthesis assay (figure 3*d*) of PBP1B or PBP1A with radioactive lipid II with and without their cognate Lpo at either pH 7.5 or 5.0, as indicated. The resulting PG was digested with muramidase (cellosyl) yielding muropeptides, which were reduced with sodium borohydride and separated by HPLC. Radioactivity scale bars correspond to 500 cpm. Peak 1 corresponds to monophosphorylated disaccharide pentapeptide, peak 2 to disaccharide tetrapeptide, peak 3 to disaccharide pentapeptide, peak 4 to bis-disaccharide tetratetrapeptide, peak 5 to bis-disaccharide tetrapentapeptide and peak 6 to tris-disachharide tetratetrapentapeptide [107]. Peaks 2 and 4 are the result of DD-CPase activity, and are highlighted in red.

We have now observed CPase activity for both major *E. coli* synthases, PBP1A and PBP1B, along with their previously reported GTase and TPase activities [103,104]. CPase products were significantly enhanced when PBP1A and PBP1B were assayed with lipid II substrate in the presence of their cognate Lpo protein, and at a mild acidic pH of 5.0 (peaks 2 and 4; figure 4). Peak 2 corresponds to the monomeric disaccharide tetrapeptide, and peak 4 to the dimeric bisdisaccharide tetratetrapeptide. Both these muropeptides arise from DD-CPase activity and their formation is blocked by the specific inhibition of PBP1A or PBP1B TPase with cefsulodin (figure 4), which does not inhibit class C PBPs.

DD-CPase activity is normally associated with PG remodelling hydrolases such as PBP5 [108]. These enzymes are thought to play a role in regulating cell morphogenesis through limiting the availability of pentapeptides as donor substrates for TPase reactions, thus perhaps regulating the extent of cross-linkage in the PG. Here, we propose that the class A PBPs are capable of removing a tetrapeptide donor peptide bound to its active site serine residue by CPase reactions. Such an ‘escape’ mechanism would be required to resolve the acyl-enzyme complex if an acceptor peptide is not available. In such a situation, an unresolved acyl-enzyme complex between the TPase active site and a donor peptide would result in enzyme inhibition analogous to inhibition by a β-lactam antibiotic. Hence, the CPase activity of class A PBPs could release a donor peptide in the absence of an acceptor, and this could be particularly important when the enzyme's TPase is stimulated with increased donor binding, as might occur in the presence of Lpo proteins (see section ‘Regulation of PBP activity’).

## 9. Class A penicillin-binding proteins are active when reconstituted in a membrane

**Figure 5.**
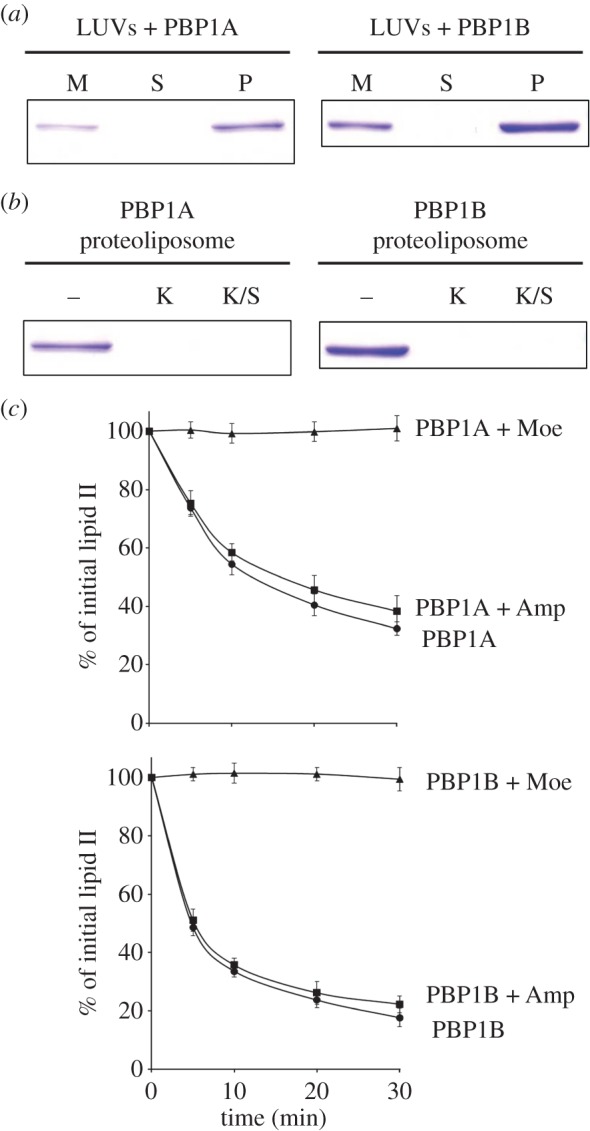
Activity of PBP1A and PBP1B in a membrane environment. (*a*) Incorporation of PBP1A and PBP1B into LUVs made of *E. coli* total lipid mixture. Samples were taken at various stages of proteoliposome preparation and resolved by SDS–PAGE followed by Coomassie blue staining. M, sample of the mixture of LUVs and purified protein; S, sample of the supernatant resulting from centrifugation of the mixture after detergent removal (Biobead treatment); P, LUVs with PBP. (*b*) Proteinase K digests PBP1A and PBP1B present in LUVs, suggesting that the PBPs were oriented outward. Untreated (–), proteoliposomes were pelleted by centrifugation without proteinase K treatment; treated (K), proteoliposomes were incubated with proteinase K prior to centrifugation; SDS extract (K/S), proteoliposomes were disrupted by SDS after proteinase K treatment to release any protein that was facing the interior of the LUV. All LUV-bound protein was accessible for proteinase K digestion, and no protein was detected in the interior of the LUVs. (*c*) Consumption of [^14^C]lipid II by PBP1A or PBP1B over time in LUVs. Amp, ampicillin; Moe, moenomycin.

Class A PBPs are integral membrane proteins and must be active in the lipid bilayer of the cytoplasmic cell membrane. To mimic such a lipidic environment, we have established a protocol to reconstitute *E. coli* PBP1A and PBP1B in proteoliposomes (large unilamellar vesicles, LUVs) containing polar phospholipids from *E. coli* (figure 5*a*). These proteoliposomes contain the PBP molecules facing to the outside, as demonstrated by the quantitative degradation by proteinase K, which can only access outside-oriented protein (figure 5*b*). Hence, the orientation of PBP in these liposomes is homogeneous and the same as in the cytoplasmic membrane.

We next tested whether PBP1A and PBP1B present in proteoliposomes were active in polymerizing radiolabelled lipid II, which was added from the outside and inserted into the liposomes. For this, samples were withdrawn after several periods of time, and extracted by butanol/pyridine, which does not extract the PG polymer, and quantified. Hence, the assay measures the consumption of the radiolabelled lipid II over time. Indeed, we could observe lipid II consumption for both enzymes reconstituted into the liposomes (figure 5*c*). As expected, the presence of ampicillin had no effect on lipid II consumption by either enzyme, and moenomycin completely inhibited both of them.

## 10. Transpeptidase activity of class B penicillin-binding proteins

*Escherichia coli* has two essential class B PBPs, PBP2 and PBP3, which participate in and are required for cell elongation and cell division, respectively. Cells depleted of PBP2 become spherical owing to their inability to elongate, and cells depleted of PBP3 grow filamentous as cell division is inhibited [109]. In class B PBPs, the N-terminal membrane anchor is linked to a non-catalytic domain that functions as a ‘pedestal’ to place the C-terminal TPase domain away from the cell membrane and near the PG layer [110] (figure 1*b*). In PBP3 and presumably other class B PBPs, the pedestal domain also stabilizes the protein, is involved in the dimerization of the protein and probably interacts with other proteins [111,112]. In *E. coli*, PBP2 interacts with PBP1A and PBP1B interacts with PBP3 [48,113]. The crystal structures of several class B PBPs are known, including the recently published structure of *E. coli* PBP3 [112].

The TPase domain is similar in amino acid sequence and, where known, structure to those of class A PBPs [4]. However, to observe an activity for a class B PBP with a natural PG substrate has proved to be difficult. Recently, for the first time, a TPase activity of a class B PBP was shown for *E. coli* PBP2 [113]. Purified PBP2 bound the β-lactam antibiotic bocillin and bocillin binding was inhibited by pre-incubating PBP2 with the specific antibiotic mecillinam, indicating that the TPase domain was folded and active. However, the purified enzyme alone did not cross-link lipid II. PBP2 was also not active in the presence of a TPase-inactive version of its interaction partner, class A PBP1A, which was capable of synthesizing glycan strands, indicating that ongoing glycan strand polymerization of PBP1A is not sufficient to stimulate TPase activity of PBP2 [113]. PBP2 stimulated PBP1A's GTase activity in different assays. PBP1A is capable of attaching a fraction of newly synthesized PG (from lipid II) to sacculi *in vitro* [104]. Interestingly, the presence of PBP2 doubled the amount of the attached material, and experiments using specific inhibitors for PBP1A (cefsulodin) and/or PBP2 (mecillinam) proved that PBP2 contributes to the attachment of new PG to sacculi by virtue of its TPase activity [113]. Hence, PBP2 requires ongoing PG synthesis by PBP1A and PG sacculi for activity, illustrating the complex regulation and specificity of this enzyme. Presumably, within the PBP1A–PBP2 complex, the pentapeptides present in nascent glycan strands produced by the GTase domain of PBP1A are used by the TPase domains of both PBPs with acceptor peptides present in the sacculi, attaching the new strand to the sacculi [113]. Such reactions must occur in a growing cell, where new PG is attached to the existing sacculus by TPase reactions [114,115]. Similar to what we have described for *E. coli* PBP2, it was recently shown that the class B PBPs from *S. pneumoniae*, PBP2b and 2x, were active in TPase reactions in the presence of a class A PBP (PBP2A) with active GTase but mutationally inactive TPase domain [75]. Unlike *E. coli* PBP2, the pneumococcal class B PBPs did not require the presence of high-molecular weight PG for TPase activity.

*Escherichia coli* PBP3 is active with thioester substrates, either performing hydrolysis or transpeptidation with d-alanine as acceptor [96,116]. However, to the best of our knowledge, no TPase activity of PBP3 with a natural substrate has been reported. In our hands, we were unable to observe TPase of PBP3 with lipid II or polymerized glycan strands in the presence or absence of PBP1B (ongoing GTase reactions), FtsN (see below) and/or PG sacculi, despite the ability of the purified PBP3 to bind β-lactam antibiotics indicating a proper fold of the TPase domain (unpublished data). Hence, it remains a conundrum what activates PBP3 in the cell. Possibly, PBP3 requires one or more yet unknown interaction partners within the divisome (such as FtsQLB or FtsW), or the particular membrane and PG architecture at the tip of the septum for activity.

## 11. Lipid II structure affects TPase activity

In many species, lipid II becomes modified prior to its polymerization. Many Gram-positive bacteria amidate the *α*-carboxylic group of the iso-glutamic acid residue at position 2 of the peptide by the amidotransferase MurT/GatD, which was identified in *S. aureus* [73,74]. Other species such as *B. subtilis*, *Lactobacillus* and Corynebacteriales amidate the ε-carboxylic group of *m*-Dap by the enzymes AsnB1 and LtsA, respectively [117,118]. Another modification is the attachment of amino acid branches by Fem transferases which, upon TPase reactions, lead to inter-peptide bridges [119].

Recent data show that the presence of modifications on lipid II affect TPase activity. *S. pneumoniae* PBP2a did not show TPase activity when using an unamidated lipid II [95], but PBP2a and other pneumococcal PBPs (PBP1a, PBP2b and PBP2x) showed TPase activity with the amidated lipid II [75]. This indicates that lipid II amidation is a requirement for TPase activity. In contrast, *B. subtilis* PBP1 was capable to perform TPase reactions, albeit to low extent, with both amidated and unamidated lipid II [97].

## 12. Regulation of penicillin-binding protein activity

Bacteria regulate PG synthesis at multiple levels to balance PG growth with the synthesis of other cell envelope layers and, in general, with cell growth. One important aspect might be the regulation of the synthesis rate and flux of the precursor lipid II, which has to be flipped across the cytoplasmic membrane to reach the PG synthases. The nature of the flippase is currently under debate. Purified FtsW/RodA flip lipid II in liposomes [72,120], consistent with their essentiality for cell division or elongation, their cellular localization and their interactions with PG synthases [8]. Based on more indirect *in vivo* data, MurJ was recently suggested to be the lipid II flippase instead of FtsW [121], although purified MurJ does not exhibit flippase activity [120].

**Figure 6.**
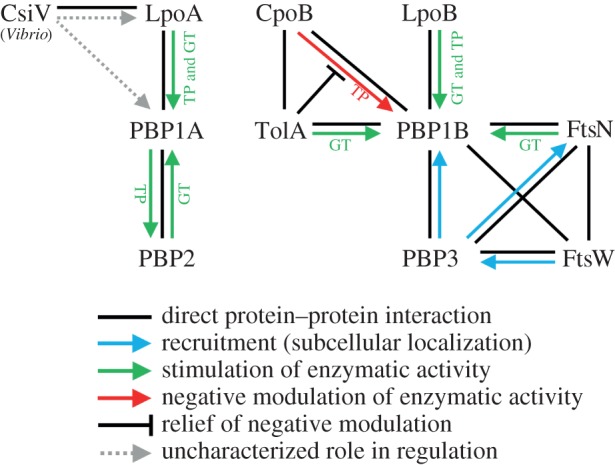
Regulation of PG synthases in *E. coli* through protein–protein interactions. Scheme of the interactions of the major PG synthases of *E. coli* and their effects on enzyme activities and cellular localization. This figure is supplementary to table S1 which contains the references. An example of regulation in *Vibrio cholerae* which is not found in *E. coli* is also shown, distinguished by (*Vibrio*). Black lines: direct interaction. Blue arrow: recruitment to subcellular location, with the direction indicating the protein recruited. Green arrow: stimulatory effect with the direction indicating the affected synthase, the particular affected activity (GT and/or TP) is also indicated. Red arrow: negative modulation of PBP1B-LpoB TPase by CpoB, which is reversed by TolA as indicated by the capped black line. Grey-dashed arrow: uncharacterized role in regulation in the cell.

Table 1 and figure 6 summarize factors that affect the activities of PG synthases. An important aspect of the regulation of PG synthases are protein–protein interactions, of which several are known to directly affect enzyme activities and subcellular localization (figure 6). Here, we mainly focus on the PG synthases of *E. coli* as these are the most studied.

**Table 1.** Known factors affecting the activity or regulation of PG synthases.

category	examples^a^ with references
protein–protein interaction	*E. coli* **PBP1A**—LpoA — Essential for PBP1A function in the cell [106,122]. — Interaction stimulates the TPase activity: cross-links in PG product increases by 20% [106]; TPase rate (d-Ala incorporation) increases sixfold with concomitant 1.5-fold increase in GTase rate [98]. *E. coli* **PBP1A**—PBP2 [113] — Interaction stimulates the GTase reaction rate of PBP1A approximately fivefold and increases the mean glycan length. — Interaction stimulates the TPase activity of PBP1A resulting in more efficient attachment of newly synthesized PG to sacculi. *E. coli* **PBP1B—**dimerization [103] — Significantly stimulated GTase and TPase activities. *E. coli* **PBP1B**—LpoB — Essential for PBP1B function in the cell [106,122,123]. — Interaction stimulates the GTase approximately eightfold [123] and reduces the mean glycan chain length [98]. — Interaction stimulates the TPase resulting in 20% more cross-links in the PG product [106] and a 1.5-fold increase in d-Ala incorporation rate [98]. *E. coli* **PBP1B**—FtsN — Interaction stimulates PG synthesis activities of PBP1B at low concentration, possibly by promoting dimerization of PBP1B [124]. — Stimulation of the GTase rate approximately fourfold, acts synergistically with LpoB (this work). *E. coli* **PBP2**—PBP1A [113] — Interaction stimulates the TPase activity of PBP2 for the attachment of newly synthesized PG to sacculi. *E. coli* **PBP1B-LpoB**—CpoB/TolA [125] — Interaction of CpoB partially prevents stimulation of TPase by LpoB, with a 50% reduction in stimulation. — Interaction of TolA reverses the effect of CpoB on TPase and also stimulates GTase approximately 1.9-fold, acts synergistically with LpoB. *V. cholerae* **PBP1A-LpoA**—CsiV [126] — Δ*csiV* phenocopies Δ*lpoA* and Δ*mrcA*. — CsiV interacts directly with LpoA and is essential for PBP1A function in the cell when grown in the presence of 5 mM d-Met.
localization or spatial regulation	*E. coli* **PBP1B**—PBP3 [48] — PBP1B requires PBP3 for septal localization. *E. coli* **PBP3**—FtsW [127] — PBP3 requires FtsW for septal localization. *B. subtilis* **PBP1**—GpsB/EzrA [128] — PBP1 requires GpsB/EzrA for relocation from the side wall to the septum. — GpsB removes PBP1 from new cell pole post-division.
precursor/substrate	*S. pneumoniae* **PBP1a, PBP2a, PBP2b,** and **PBP2x**—amidation of lipid II [75] — TPase activity requires the presence of an amidated iso-Gln residue at position 2 of the stem peptide. *S. aureus* **MtgA**—lipid II [86] — Binding of lipid II enhances the affinity of moenomycin to the glycan acceptor site.
environmental conditions	*C. crescentus* **PBP2** and **PBP1A**—osmolarity of growth medium [129] — Upshift in the osmolarity of growth medium enhances localization to mid-cell relocating the PG growth site. *E. coli* **PBP1B** and **PBP1A**—pH — GTase activity is reduced at pH 4.5^b^ [123]. — enhanced CPase activity at pH 5.0 (this work).

^a^Synthase shown in bold, regulator/effector underlined.

^b^Only experimentally shown for PBP1B.

### (a) Outer membrane lipoproteins are required for the cellular function of class A PBPs

In *E. coli*, it was recently found that both major PG synthases require cognate OM lipoproteins for function in the cell. LpoA and LpoB are essential for the activities of PBP1A and PBP1B, respectively [106,122]. The cell requires either PBP1A-LpoA or PBP1B-LpoB for growth, with the depletion of one of the *lpo* genes in the absence of the other resulting in cell lysis, mirroring the phenotype of their cognate PG synthase mutants [106,122,130]. The Lpo proteins activate their cognate PBP by direct interaction with a specific docking domain. The Lpo proteins also show the same preference for subcellular localization as their cognate PBP. PBP1A and LpoA preferentially localize to the side wall, and PBP1B and LpoB localize to the side wall and are enhanced at the division site. However, in contrast to its cognate PBP, the localization of LpoB requires the activity of PBP3 as it is diminished in cells treated with the PBP3-specific β-lactam aztreonam, presumably because LpoB localization requires ongoing septal PG synthesis [106].

LpoB was shown to interact with a small non-catalytic domain within PBP1B, called UB2H, situated between the GTase and TPase domains (figure 1*b*), via a relatively large interface [123]. The structure of full-length LpoB was solved by NMR spectroscopy [123]. LpoB has a small globular C-terminal domain, within which is the interaction site for PBP1B, and a long proline-rich unstructured N-terminal region. This flexible, disordered region has a maximal length of 145 Å allowing LpoB to reach from the OM to interact with and activate its cognate synthase [123]. The structure of the globular domain of LpoB from both *E. coli* and *Salmonella enterica* was also solved by X-ray crystallography [131].

LpoA is larger and more rigid than LpoB and does not rely on a long flexible region to reach from the OM to its cognate synthase. Instead, LpoA adopts an elongated fold with two distinct domains [132]. The structure of the N-terminal domain of LpoA was solved by NMR spectroscopy, and found to comprise a series of 5 helix-turn-helix tetratricopeptide-repeat (TPR)-like motifs. TPR motifs are protein–protein interaction modules implicated in multiprotein complex formation and are found in all kingdoms of life [133,134]. The C-terminal domain of LpoA from *E. coli* has two extended flexible regions of unknown function that presumably prevent crystallization [132]. However, the structure of the C-terminal domains from *Haemophilus influenzae* LpoA, which lacks these regions, was solved showing similarity to periplasmic binding protein domains [135]. SAXS, AUC and NMR data of full-length *E. coli* LpoA suggest that there is no flexibility between the N- and C-terminal domains, and that the overall shape of the molecule is extended, giving it a length of approximately 140–150 Å [134]. This distance would be sufficient for LpoA to reach from the OM to the inner membrane (IM)-anchored PBP1A. PBP1A contains a small non-catalytic domain, called ODD, which co-occurs with LpoA in the γ-proteobacteria. The overexpression of isolated ODD domain into the periplasm of *E. coli* lacking PBP1B or LpoB, therefore reliant on PBP1A-LpoA for growth, caused lysis [106]. This suggested that the expressed ODD competed with PBP1A for binding to LpoA and, hence, that ODD is the docking domain for LpoA [106]. Analysis of the primary sequence of class A PBPs from several species revealed the presence of amino acid regions outside the catalytic domains. These were often specific to closely related group(s) of bacteria, and it was hypothesized that many of these regions are docking domains for regulatory inputs [8].

### (b) Activation of penicillin-binding proteins by Lpo proteins

Both Lpo proteins directly affect the PG synthesis activities of their cognate PBP *in vitro*. In both cases, the TPase activity is stimulated, increasing the percentage of peptides in cross-links by approximately 20% when using lipid II as substrate [106]. LpoB also reduces the length of the glycan strands produced by PBP1B (in the absence of TPase reactions). However, these end-point assays measured the product formed, and could not determine any possible change in TPase or GTase rate. Subsequently, it was shown that Lpo proteins increase the TPase rate of their cognate PBP using the incorporation of radiolabelled d-Ala as proxy for TPase activity [98]. The GTase and TPase activities of class A PBPs are coupled, with TPase activity dependent upon ongoing GTase (discussed above), raising the following question: do the Lpo proteins stimulate one enzymatic activity which concomitantly increases the other, or do they affect both simultaneously? This remains largely unclear and recent data suggests that the primary stimulatory mechanism differs between LpoA and LpoB [98].

In addition to enhancing TPase activity, LpoA was shown to mildly enhance the rate of PBP1A GTase activity approximately 1.5-fold using the d-amino acid incorporation assay (figure 3*d*) [98]. Interestingly, this stimulation is blocked by addition of penicillin G, suggesting that the GTase stimulation requires enhanced TPase activity [98]. Using this assay LpoB was also shown to enhance PBP1B GTase approximately 1.5-fold, but this was not blocked by penicillin G. Thus, the authors suggest that LpoA primarily affects the TPase activity of PBP1A, and LpoB the GTase activity of PBP1B, which concomitantly affects the other domain [98]. Using the more sensitive continuous fluorescence assay and dansyl lipid II as substrate, LpoB's stimulation of the PBP1B GTase activity was shown to be approximately eightfold and independent of TPase reactions [123]. In this assay, LpoB could rescue PBP1B GTase activity at a pH of 4.5, at which the enzyme alone was virtually inactive. Thus, we hypothesize that LpoB-binding to the UB2H domain of PBP1B may cause an allosteric effect on the GTase domain, inducing conformational change within the GTase catalytic site leading to activation [123]. However, our current understanding of the regulatory mechanisms of the Lpo proteins remains incomplete owing to limitations of the available assays and the lack of co-structures of the complexes.

### (c) The cell division protein FtsN stimulates PBP1B GTase activity

FtsN is an essential division protein recruited to mid-cell prior to the onset of constrictive PG synthesis [136]. It interacts with other essential division proteins FtsA, FtsQ, PBP3 and FtsW (summarized in reference [137]). FtsN is a bitopic membrane protein with a short cytoplasmic region, a single transmembrane helix and a flexible periplasmic region which features three *α*-helices followed by a proline/glutamine-rich unstructured region and a globular C-terminal SPOR domain which binds to PG but is not essential [138,139]. Extensive mutagenesis showed that only three amino acid residues (W83, Y85 and L89) in the periplasmic part are critical for FtsN function [140]. FtsN may be involved in the transduction of a signal from the late to the early divisome, to begin cytokinesis after the maturation of the complex. Mutations in *ftsQLB and ftsA* could bypass the need for FtsN, and the altered proteins acted synergistically to restore cell division in its absence [140].

Different versions of purified FtsN interacted with PBP1B, including full-length FtsN, a soluble version lacking the cytoplasmic and transmembrane region, and several truncations of this soluble version, suggesting that there are several interaction sites [124]. Interestingly, the full-length protein containing the transmembrane and cytoplasmic region stimulated the PG synthesis activity of PBP1B at conditions where it did not dimerize and was poorly active. Hence, we hypothesized that FtsN is capable of promoting dimerization of PBP1B, enhancing its activities [103,124].

**Figure 7.**
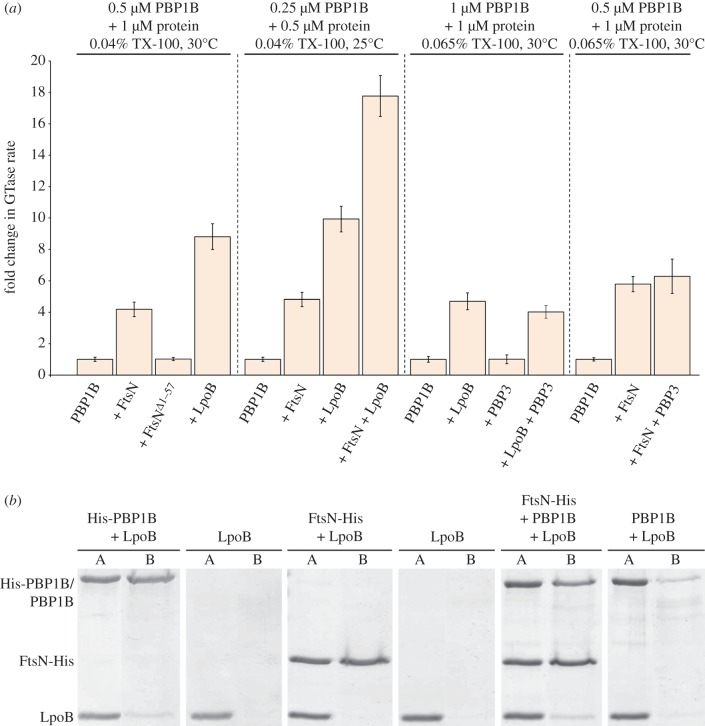
LpoB and FtsN synergistically enhance the GTase activity of PBP1B. (*a*) GTase activity of PBP1B was assayed by consumption of fluorescently labelled lipid II *in vitro*. Change in GTase rate is relative to PBP1B alone at the indicated reaction conditions and is shown as the mean ± s.d. (*n* = 4–12). Reaction conditions (Triton X-100 (TX-100) concentration, temperature and enzyme concentration) were optimized for each experiment. Specific conditions are indicated above the corresponding data. (*b*) A ternary complex of FtsN-PBP1B-LpoB was detected by *in vitro* cross-linking/pulldown approach. Proteins were cross-linked and applied to Ni-NTA beads. Cross-linkage of bound proteins was cleaved and samples separated by SDS–PAGE and visualized with Coomassie blue. FtsN-His retained LpoB only in the presence of PBP1B. The relatively weak retention of LpoB by PBP1B also occurs in the absence of FtsN, using His-PBP1B instead. This may be due to poor cross-linking efficiency between PBP1B and LpoB. A, applied sample; B, retained/bound protein.

We have now used the continuous GTase assay with dansyl-lipid II to assess the effect of FtsN on PBP1B activity. Full-length FtsN-His stimulated the GTase activity of PBP1B 4.2 ± 0.5-fold (figure 7*a*). Consistent with our previous data [124], FtsN lacking the transmembrane and cytoplasmic domains (FtsN^Δ1–57^-His) had no effect, and FtsN-His alone had no effect on the fluorescent lipid II (figure 7*a*). Interestingly, the stimulation of the GTase of PBP1B by FtsN was synergistic with the stimulation by LpoB (figure 7*a*). With FtsN and LpoB, the GTase rate increased 16.9 ± 0.9-fold, more than with either LpoB (9.3 ± 0.9-fold) or FtsN (4.6 ± 0.4-fold) alone. Because it was possible that two subpopulations of enzymatically active complexes exist within the reaction (PBP1B-LpoB and PBP1B-FtsN), we sought to determine whether LpoB and FtsN are able to interact simultaneously with PBP1B in a pull-down assay using FtsN-His with PBP1B and LpoB, exploiting the fact that LpoB and FtsN do not interact directly. Indeed, untagged LpoB (soluble version) was retained by FtsN-His on Ni-beads only in the presence of PBP1B (figure 7*b*), suggesting that both regulators bind to PBP1B simultaneously to exert a synergistic effect on activity.

Together, these data suggest that FtsN ensures coordination of PG synthesis with cytokinesis through multiple interactions with PG synthases and cell division proteins and a direct stimulation of PBP1B activity.

### (d) PBP3 has no effect on the glycosyltransferase activity of PBP1B

FtsN interacts with both PBP1B and PBP3, and the synthases also interact directly with each other [48]. Here, we tested whether PBP3 may play a role in the regulation of the GTase activity of PBP1B. Using the continuous GTase assay with dansyl-lipid II as substrate, we found that PBP3 had no effect on PBP1B GTase activity directly, nor did it impact the stimulation by FtsN (figure 7*a*). Additionally, His-PBP3 did not have an effect on the stimulation of PBP1B by LpoB (figure 7*a*).

### (e) Coordination of peptidoglycan synthesis with outer membrane constriction in *Escherichia coli*

Recently, the stimulation of PBP1B by LpoB was found to be modulated by proteins of the Tol system [125]. The Tol system in *E. coli* features three IM-anchored proteins, TolA, TolQ and TolR, which form a complex [141]. TolQ and TolR are able to harness the proton motive force (pmf) to energize TolA, driving conformational changes in its periplasmic domains, which is required for function [142]. The Tol system also includes a periplasmic protein, TolB, which interacts with the C-terminal domain of TolA and with the abundant PG-binding OM lipoprotein Pal [143], the final core member of the system. Depleting the cell of any of the five components leads to a *tol–pal* phenotype, typically exhibiting as severe defects in OM stability and a delayed onset of constriction during cell division [144]. Consistent with this, each of the core Tol proteins localizes to mid-cell during cell division dependent on the divisome complex [144].

TolA interacts with CpoB (formerly YbgF) [145] whose cellular role was unclear, as a *cpoB* mutant did not show a *tol–pal* phenotype. Recently, both TolA and CpoB were shown to interact directly with PBP1B-LpoB *in vitro* and in the cell [125]. Additionally, CpoB was found to localize to mid-cell at the onset of constriction, requiring a functional divisome complex. CpoB binds to PBP1B between the UB2H and TPase domains. Consistent with this binding site, CpoB partially inhibits the stimulation of the TPase activity of PBP1B by LpoB *in vitro*. Remarkably, this effect of CpoB is relieved by TolA, which interacts with PBP1B at the region proximal to the membrane. TolA alone or with CpoB moderately enhances the GTase of PBP1B (1.9 ± 0.5-fold), and this effect is synergistic with the stronger stimulation by LpoB. Furthermore, the interactions of TolA and CpoB with PBP1B-LpoB are responsive to the assembly of the Tol complex and its energy state in the cell. No direct interaction was detected between CpoB and LpoB either *in vitro* or in wild-type cells. However, in the absence of *tolA*, *tolQ* or *tolR* a strong association between CpoB and LpoB is seen in the cell, probably through PBP1B. This is also seen in a strain with a point mutation in *tolR* preventing TolQRA from harnessing the pmf. Thus, the modulation of the TPase of PBP1B-LpoB by TolA and CpoB depends on the state of the Tol system, and the functional link between these two envelope-spanning machineries coordinates PG synthesis with OM constriction during cell division [125].

### (f) CsiV regulates PBP1A-LpoA in *Vibrio*

The regulation of PBP1A by LpoA in *Vibrio cholerae* involves an additional factor [126], the small, periplasmic protein CsiV, which was discovered by a chemical synthetic lethal screen. A *csiV* deletion closely phenocopied *mrcA* (encoding PBP1A) and *lpoA* deletions, and CsiV interacted directly with LpoA in the cell. *LpoA*, *mrcA* and *csiV* are essential for growth in the presence of 5 mM d-methionine. These strains also have an altered PG content, particularly in stationary phase. However, while evidence suggests CsiV is important for the function of PBP1A-LpoA it is not strictly essential; a *csiV lpoB* double mutant, which relies on PBP1A-LpoA, is viable. Thus, the precise role of CsiV remains to be determined [126].

### (g) Regulation of penicillin-binding protein localization

*Escherichia coli* PG synthases interact with several cell morphogenesis proteins and regulators (summarized in references [8,137]), and some of these interactions appear to be required to localize PBPs to the elongasome and divisome, respectively. For example, PBP3 and PBP1B interact in non-dividing cells [48], and PBP3 is recruited (presumably together with PBP1B) to the divisome by interactions with FtsQLB and FtsW. In other species, there can be variations in the interactions and the divisome recruitment pathway (e.g. in *Caulobacter crescentus* [146]).

The Gram-positive *B. subtilis* has additional factors to the system of distinct, cytoskeleton controlled, elongation and division complexes for spatial regulation of PG synthesis. EzrA and GpsB control the localization of the major PG synthase PBP1 during the cell cycle [128]. EzrA is the first example of a bacterial spectrin-like protein, with an arc-like structure, which interacts with both FtsZ and FtsA [147]. EzrA contributes to membrane-anchoring of FtsZ, regulating its dynamics. Cells lacking EzrA formed aberrant, multiple Z-rings and showed a delay in division [148]; a *gpsB ezrA* double mutant has more severe cell division defects with aberrant bulges at cell poles and division sites. A network of interactions between PBP1 and GpsB, MreC (MreB-associated protein) and EzrA was observed by bacterial two-hybrid assays, indicating that PBP1 interacts with cell elongation and division proteins consistent with its localization pattern [128]. GpsB functions to recruit PBP1 from the elongation to the division complex and also removes PBP1 from the new cell poles after division, making it available for elongation, a function which is seemingly crucial in the *B. subtilis* cell cycle [128].

In some species, the cellular localization of PG synthases can also be regulated by environmental conditions. In *C. crescentus*, even small osmotic upshifts cause PBP1A and PBP2 to relocate, moving from a patchy side wall location to the position of FtsZ at mid-cell [129]. While the relocation of PBPs occurs within minutes, the restoration of their normal side wall localization pattern requires cell growth and takes one to two generations. This phenomenon appears to be specific for *C. crescentus*, as the localization of PBPs in *E. coli* was largely unaffected by an osmotic up-shift [129].

## 13. Concluding remarks

How bacteria synthesize PG and expand their sacculus to grow and divide, and how the process is regulated and coordinated with the synthesis of other cellular components, have remained highly fascinating but yet unanswered questions in microbiology. Nonetheless, the past decade brought substantial increase in our knowledge of PG synthesis, which was made possible by the improvement of tools available, like the different lipid II versions and novel *in vitro* PG synthesis assays. Major recent advances were the discoveries of interactions between PG synthases and other proteins that influence GTase and/or TPase activities. We are beginning to get an idea of the multiplicity and complexity of PG synthesis regulation. With further technical advances and increasing knowledge of all the components involved we should be able to dissect the molecular mechanisms of bacterial cell wall growth. We hope that understanding this fundamental process will also help to identify novel targets for antimicrobial drug discovery.

## 14. Material and methods

### (a) Chemicals and proteins

[^14^C]Glc*N*Ac-labelled lipid II and dansylated lipid II were prepared as published [63,103]. The following proteins were prepared as previously described: PBP1B [48], PBP1A [104], LpoB(sol) [123]. PBP3, FtsN and FtsN^Δ1–57^ were overproduced using previously published strains and plasmids [48,139] with modifications to the purification procedure (below).

### (b) Protein overproduction and purification

*FtsN-His.* Cells of BL21(DE3) pFE42 [139] were grown in 2 l of LB medium with 100 µg ml^−1^ ampicillin at 37°C to an OD_578_ of 0.4. FtsN-His was overproduced by adding 1 mM IPTG to the cell culture followed by a further incubation for 2 h at 37°C. Cells were harvested by centrifugation (10 000*g*, 15 min, 4°C), and the pellet was resuspended in buffer I (25 mM Tris/HCl, 1 M NaCl, pH 6.0). A small amount of DNase, protease inhibitor cocktail (Sigma, USA, 1/1000 dilution) and 100 µM phenylmethylsulfonylfluoride (PMSF) was added before cells were disrupted by sonication (Branson Digital, USA). The lysate was centrifuged (130 000*g*, 1 h, 4°C). The resulting membrane pellet was resuspended in extraction buffer (25 mM Tris/HCl, 1 M NaCl, 40 mM imidazole, 1% Triton X-100, pH 6.0) and incubated overnight with mixing at 4°C. The sample was centrifuged (130 000*g*, 1 h, 4°C) and the supernatant applied to a 5 ml HisTrap HP column (GE Healthcare, USA) attached to an ÄKTA Prime+ (GE Healthcare, USA), at 1 ml min^−1^. The column was washed with four volumes extraction buffer, followed by four volumes of wash buffer I (25 mM Tris/HCl, 1 M NaCl, 40 mM imidazole, 0.25% Triton X-100, pH 6.0). Bound protein was eluted step-wise with elution buffer (25 mM Tris/HCl, 1 M NaCl, 400 mM imidazole, 0.25% Triton X-100, pH 6.0). FtsN-His was dialysed into storage buffer (25 mM Tris/HCl, 500 mM NaCl, 0.25% Triton X-100, 10% glycerol, pH 6.0) and stored in aliquots at −80°C.

*FtsN^Δ1–57^-His.* Cells of BL21-A1 pHis17-ECN2 [139] were grown in 2 l of LB medium supplemented with 100 µg ml^−1^ ampicillin at 30°C to an OD_578_ of 0.5. FtsN^Δ1–57^-His was overproduced by adding 0.2% arabinose to the cell culture followed by a further incubation for 3 h at 30°C. Cells were harvested by centrifugation (10 000*g*, 15 min, 4°C) and the pellet was resuspended in buffer I (25 mM Tris/HCl, 500 mM NaCl, pH 6.0). A small amount of DNase, protease inhibitor cocktail (Sigma, 1/1000 dilution) and 100 µM PMSF was added before cells were disrupted by sonication (Branson Digital, USA). The lysate was centrifuged (130 000*g*, 1 h, 4°C). The resulting supernatant was applied to 1.5 ml of Ni-NTA superflow beads (Qiagen, The Netherlands), supplemented with 10 mM imidazole and incubated for 18 h at 4°C. Beads were washed with 7 × 10 ml wash buffer (25 mM Tris/HCl, 500 mM NaCl, 20 mM imidazole, pH 6.0) and bound protein eluted with 10 × 1 ml elution buffer (25 mM Tris/HCl, 500 mM NaCl, 300 mM imidazole, pH 6.0). Appropriate fractions were pooled and dialysed into storage buffer (25 mM HEPES/NaOH, 500 mM NaCl, 10% glycerol, pH 6.0) and stored in aliquots at −80°C.

*His-PBP3.* Cells of XL1-Blue pMVR1 [48] were grown in 5 l of LB medium supplemented with 5% glycerol and 20 µg ml^−1^ chloramphenicol at 30°C to an OD_578_ of 0.6. His-PBP3 was overproduced by adding 0.05 mM IPTG to the cell culture followed by a further incubation for 18 h at 30°C. Cells were harvested by centrifugation (10 000*g*, 15 min, 4°C) and the pellet was resuspended in buffer I (25 mM HEPES/NaOH, pH 8.0) before another centrifugation step (as previous). The cell pellet was resuspended in buffer II (25 mM HEPES/NaOH, 1 M NaCl, pH 8.0) and a small amount of DNase, protease inhibitor cocktail (Sigma, 1/1000 dilution), and 100 µM PMSF was added before cells were disrupted by sonication (Branson Digital). The lysate was centrifuged (130 000*g*, 1 h, 4°C). The resulting membrane pellet was resuspended in extraction buffer (25 mM HEPES/NaOH, 10 mM MgCl_2_, 1 M NaCl, 20 mM imidazole, 2% Triton X-100, pH 8.0) and incubated overnight with mixing at 4°C. Sample was centrifuged (130 000*g*, 1 h, 4°C) and the supernatant applied to 1 ml of washed Ni-NTA superflow beads (Qiagen, The Netherlands). The sample was incubated with mixing for 4 h at 4°C. Beads were then washed with 3 × 10 ml wash buffer I (25 mM HEPES/NaOH, 10 mM MgCl_2_, 1 M NaCl, 20 mM imidazole, 0.2% Triton X-100, pH 8.0), followed by 4 × 10 ml wash buffer II (as wash buffer I, with 40 mM imidazole and 10% glycerol). Bound protein was eluted with 10 × 1 ml elution buffer (25 mM HEPES/NaOH, 10 mM MgCl_2_, 1 M NaCl, 400 mM imidazole, 10% glycerol, 0.2% Triton X-100, pH 8.0). Appropriate fractions were pooled and dialysed into storage buffer (25 mM HEPES/NaOH, 10 mM MgCl_2_, 1 M NaCl, 10% glycerol, 0.2% Triton X-100, pH 8.0) and stored in aliquots at −80°C. The purified His-PBP3 was able to bind the fluorescent β-lactam bocillin (Molecular probes, USA) suggesting correct folding of the TPase domain (not shown).

### (c) Preparation of proteoliposomes wih PBP1A and PBP1B

LUVs containing PBP1A or PBP1B were prepared according to previously described methods with modifications [149–151]. A total of 10 mg *E. coli* total lipid mix (Avanti Lipids, USA) was dried in a glass test tube under a stream of nitrogen gas. The resulting lipid film was further dried under vacuum in a desiccator for 2 h. Lipids were then rehydrated to a concentration of 10 mg ml^−1^ by the addition of 1 ml of 20 mM HEPES/NaOH, 100 mM NaCl pH 7.5. The resulting hydrated lipids are mainly in the form of multilammellar vesicles. The vesicles were freeze–thawed 10 times using liquid nitrogen and a water bath set at 42°C and were then extruded 10 times through a 0.2 µm Anatop-10 inorganic membrane filter (Whatman (GE Healthcare), USA). At this point, nearly all the vesicles present were unilamellar vesicles [150]. The size of the LUVs formed was confirmed by dynamic light scattering (DLS) using a Zetasizer instrument (Malvern Technologies, UK). PBP1B or PBP1A purified in the presence of Triton X-100 and with a final concentration of 1.5 µM was added to 350 µl LUVs and incubated for 1 h at 4°C with rotary mixing. Wet prewashed Biobeads SM2 (100 mg; BioRad, USA) were added to the sample. Biobeads were then exchanged after 2 and 16 h, followed by incubation with fresh Biobeads for a further 2 h. Biobeads were removed by centrifugation at 4000*g*, and the supernatant was centrifuged at 250 000*g* for 30 min at 4°C. The resulting pellet containing the PBP-proteoliposomes was resuspended in 200 µl of 20 mM HEPES/NaOH, 100 mM NaCl, pH 7.5. Incorporation of the PBPs into LUVs was tested by comparison between the pellet and supernatant after the 250 000*g* centrifugation by SDS–PAGE, with Coomassie blue staining (figure 5*a*). To test the orientation of the proteins within the proteoliposomes, the samples were treated with 0.5 µg µl^−1^ proteinase K for 15 min at 37°C followed by centrifugation to pellet the LUVs. The resulting pellets were boiled in SDS–PAGE sample buffer and analysed by SDS–PAGE, following by staining with Coomassie blue. A negative control featured disruption of the LUVs with 0.5% SDS prior to proteinase K treatment. This experiment showed that the PBPs are attached almost exclusively to the outer leaflet of the LUV bilayer (figure 5*b*).

### (d) Penicillin-binding protein activity assays in detergent solution and proteoliposomes

The *in vitro* PG synthesis assay for the observation of CPase activity of PBP1A and PBP1B was performed as previously described [152], except that 10 mM sodium phosphate, pH 5.0, was used in place of HEPES/NaOH, pH 7.5 in the reaction buffer for samples tested at this pH; all other components remained the same. Continuous fluorescence GTase assays using dansylated lipid II were performed as described previously [113] whereby the reaction conditions (Triton X-100 concentration, temperature and enzyme concentration) were varied as indicated in figure 5*a*. The lipid II consumption assay on LUVs was performed as follows. Samples consisted of 1 µM of either PBP1B or PBP1A in LUVs (comprising approx. 2.5 mg of lipids) with 5 nM [^14^C]Glc*N*Ac-labelled lipid II (15 000 dpm), 0.01% EtOH, 5 µM MgCl_2_, 100 mM NaCl in a final volume of 540 µl. Samples were incubated at 37°C in a thermal microfuge tube shaker at 800 r.p.m. Aliquots of the reaction mix (95 µl) were taken at 0, 5, 10, 20 and 30 min. Un-reacted lipid II was immediately extracted by addition of 200 µl of a 1 : 1 mixture of butanol and 6 M pyridine–acetate, pH 4.2 [63]. Samples were mixed and centrifuged at 5000*g* for 2 min. The butanol phase (approx. 100 µl) was collected in a scintillation vial, 5 ml Ecoscint A liquid scintillation cocktail (National Diagnostics, USA) was added and the radioactivity was measured using a HIDEX 300SL β-particle scintillation detector. Where indicated, samples included 0.2 mg ml^−1^ moenomycin (Hoechst, Germany) or 0.1 mg ml^−1^ ampicillin (Sigma, USA) to inhibit GTase and TPase, respectively.

### (e) *In vitro* cross-linking/pulldown assay

Proteins were mixed at appropriate concentrations (FtsN-His and PBP1B, 1 µM; LpoB(sol), 2 µM) in 200 µl of binding buffer (10 mM HEPES/NaOH, 10 mM MgCl_2_, 150 mM NaCl, 0.05% Triton X-100, pH 7.5). Samples were incubated at room temperature for 10 min before addition of 0.2% w/v formaldehyde (Sigma, USA) and further incubation at 37°C for 10 min. Excess cross-linker was blocked by addition of 100 mM Tris/HCl, pH 7.5. Samples were applied to 100 µl of washed and equilibrated Ni-NTA superflow beads (Qiagen, The Netherlands) and incubated overnight at 4°C, with mixing. The beads were then washed with 6 × 1.5 ml wash buffer (10 mM HEPES/NaOH, 10 mM MgCl_2_, 500 mM NaCl, 50 mM imidazole, 0.05% Triton X-100, pH 7.5) and boiled in SDS–PAGE loading buffer. Beads were then removed by centrifugation and samples analysed by SDS–PAGE. Gels were stained with Coomassie brilliant blue (Roth, Germany).

## References

[1] Vollmer W, Blanot D, de Pedro MA 2008 Peptidoglycan structure and architecture. FEMS Microbiol. Rev. 32, 149–167. (10.1111/j.1574-6976.2007.00094.x) 18194336

[2] Weidel W, Pelzer H 1964 Bagshaped macromolecules—a new outlook on bacterial cell walls. Adv. Enzymol. 26, 193–232. (10.1002/9780470122716.ch5) 14150645

[3] Vollmer W, Seligman SJ 2010 Architecture of peptidoglycan: more data and more models. Trends Microbiol. 18, 59–66. (10.1016/j.tim.2009.12.004) 20060721

[4] Goffin C, Ghuysen JM 1998 Multimodular penicillin-binding proteins: an enigmatic family of orthologs and paralogs. Microbiol. Mol. *Biol. Rev.* 62, 1079–1093. 10.1128/mmbr.62.4.1079-1093.1998 PMC98940 9841666

[5] Höltje J-V 1993 ‘Three for one’- A simple growth mechanism that guarantees a precise copy of the thin, rod-shaped murein sacculus of *Escherichia coli*. In Bacterial growth and lysis: metabolism and structure of the bacterial sacculus *(eds MA de Pedro*, Höltje JV, Löffelhardt W), pp. 419–426. New York, NY: Plenum Press.

[6] Höltje J-V 1998 Growth of the stress-bearing and shape-maintaining murein sacculus of *Escherichia coli*. Microbiol. Mol. Biol. Rev. 62, 181–203. 9529891 10.1128/mmbr.62.1.181-203.1998 PMC98910

[7] Sauvage E, Kerff F, Terrak M, Ayala JA, Charlier P 2008 The penicillin-binding proteins: structure and role in peptidoglycan biosynthesis. FEMS Microbiol. Rev. 32, 234–258. (10.1111/j.1574-6976.2008.00105.x) 18266856

[8] Typas A, Banzhaf M, Gross CA, Vollmer W 2012 From the regulation of peptidoglycan synthesis to bacterial growth and morphology. Nat. Rev. Microbiol. 10, 123–136. (10.1038/nrmicro2677) PMC5433867 22203377

[9] Vollmer W, Joris B, Charlier P, Foster S 2008 Bacterial peptidoglycan (murein) hydrolases. FEMS Microbiol. Rev. 32, 259–286. (10.1111/j.1574-6976.2007.00099.x) 18266855

[10] Fleming A 1929 On the antibacterial action of cultures of a *Penicillum* with special reference to their use in the isolation of *B. influenzae*. Brit. J. Exp. Pathol. 10, 226–236.

[11] Duguid JP 1946 The sensitivity of bacteria to the action of penicillin. Ed. Med. J. 53, 401–412. PMC5283540 20999634

[12] Lederberg J 1956 Bacterial protoplasts induced by penicillin. Proc. Natl Acad. Sci. USA 42, 574–577. (10.1073/pnas.42.9.574) 16589908 PMC534253

[13] Park JT, Strominger JL 1957 Mode of action of penicillin. Science 125, 99–101. (10.1126/science.125.3238.99) 13390969

[14] Park JT, Johnson M 1949 Accumulation of labile phosphate in *Staphylococcus aureus* grown in the presence of penicillin. J. Biol. Chem. 179, 585–592. 18149992

[15] Salton MRJ, Horne RW 1951 Studies of the bacterial cell wall. 2. Methods of preparation and some properties of cell walls. Biochim. Biophys. Acta 7, 177–197. (10.1016/0006-3002(51)90017-0) 14858403

[16] Tipper DJ, Strominger JL 1965 Mechanism of action of penicillins: a proposal based on their structural similarity to acyl-D-alanyl-D-alanine. Proc. Natl Acad. Sci. USA 54, 1133–1141. (10.1073/pnas.54.4.1133) 5219821 PMC219812

[17] Wise EM Jr, Park JT 1965 Penicillin: its basic site of action as an inhibitor of a peptide cross-linking reaction in cell wall mucopeptide synthesis. Proc. Natl Acad. Sci. USA 54, 75–81. (10.1073/pnas.54.1.75) 5216369 PMC285799

[18] Martin HH 1964 Chemical composition of cell wall mucopolymer from penicillin-spheroplasts and normal cells of *Proteus mirabilis*. In *Sixth Int. Conf. of Biochemistry, 26 July–1 August 1964, New York, NY*, p. 70.

[19] Izaki K, Matsuhashi M, Strominger JL 1966 Glycopeptide transpeptidase and D-alanine carboxypeptidase: penicillin-sensitive enzymatic reactions. Proc. Natl Acad. Sci. USA 55, 656–663. (10.1073/pnas.55.3.656) 5329013 PMC224202

[20] Katz W, Matsuhashi M, Dietrich CP, Strominger JL 1967 Biosynthesis of the peptidoglycan of bacterial cell walls. IV. Incorporation of glycine in *Micrococcus lysodeikticus*. J. Biol. Chem. 242, 3207–3217. 6027794

[21] Matsuhashi M, Dietrich CP, Strominger JL 1965 Incorporation of glycine into the cell wall glycopeptide in *Staphylococcus aureus*: role of sRNA and lipid intermediates. Proc. Natl Acad. Sci. USA 54, 587–594. (10.1073/pnas.54.2.587) 5218655 PMC219708

[22] Mirelman D, Bracha R, Sharon N 1972 Role of the penicillin-sensitive transpeptidation reaction in attachment of newly synthesized peptidoglycan to cell walls of *Micrococcus luteus*. Proc. Natl Acad. Sci. USA 69, 3355–3359. (10.1073/pnas.69.11.3355) 4343965 PMC389770

[23] Mirelman D, Sharon N 1972 Biosynthesis of peptidoglycan by a cell wall preparation of *Staphylococcus aureus* and its inhibition by penicillin. Biochem. Biophys. Res. Commun. 46, 1909–1917. (10.1016/0006-291X(72)90069-1) 4259358

[24] Hammes WP 1976 Biosynthesis of peptidoglycan in *Gaffkya homari*. The mode of action of penicillin G and mecillinam. Eur. J. Biochem. 70, 107–113. (10.1111/j.1432-1033.1976.tb10961.x) 12941

[25] Hammes WP, Kandler O 1976 Biosynthesis of peptidoglycan in *Gaffkya homari*. The incorporation of peptidoglycan into the cell wall and the direction of transpeptidation. Eur. J. Biochem. 70, 97–106. (10.1111/j.1432-1033.1976.tb10960.x) 12946

[26] Taku A, Fan DP 1979 Dissociation and reconstitution of membranes synthesizing the peptidoglycan of *Bacillus megaterium*. A protein factor for the polymerization step. J. Biol. Chem. 254, 3991–3999. 108266

[27] Nguyen-Disteche M, Ghuysen JM, Pollock JJ, Reynolds P, Perkins HR, Coyette J, Salton MR 1974 Enzymes involved in wall peptide crosslinking in *Escherichia coli* K12, strain 44. Eur. J. Biochem. 41, 447–455. (10.1111/j.1432-1033.1974.tb03286.x) 4593964

[28] Pollock JJ, Nguyen-Disteche M, Ghuysen JM, Coyette J, Linder R, Salton MR, Kim KS, Perkins HR, Reynolds P 1974 Fractionation of the DD-carboxypeptidase-transpeptidase activities solubilized from membranes of *Escherichia coli* K12, strain 44. Eur. J. Biochem. 41, 439–446. (10.1111/j.1432-1033.1974.tb03285.x) 4593963

[29] van Heijenoort Y, van Heijenoort J 1980 Biosynthesis of the peptidoglycan of *Escherichia coli* K-12: properties of the *in vitro* polymerization by transglycosylation. FEBS Lett. 110, 241–244. (10.1016/0014-5793(80)80082-2) 6989635

[30] Tamaki S, Nakajima S, Matsuhashi M 1977 Thermosensitive mutation in *Escherichia coli* simultaneously causing defects in penicillin-binding protein-1Bs and in enzyme activity for peptidoglycan synthesis *in vitro*. Proc. Natl Acad. Sci. USA 74, 5472–5476. (10.1073/pnas.74.12.5472) 341159 PMC431769

[31] Ishino F, Tamaki S, Spratt BG, Matsuhashi M 1982 A mecillinam-sensitive peptidoglycan crosslinking reaction in *Escherichia coli*. Biochem. Biophys. Res. Commun. 109, 689–696. (10.1016/0006-291X(82)91995-7) 6297485

[32] Maass D, Pelzer H 1981 Murein biosynthesis in ether permeabilized *Escherichia coli* starting from early peptidoglycan precursors. Arch. Microbiol. 130, 301–306. (10.1007/BF00425944) 7036928

[33] Martin HH 1984 *In vitro* synthesis of peptidoglycan by spheroplasts of *Proteus mirabilis* grown in the presence of penicillin. Arch. Microbiol. 139, 371–375. (10.1007/BF00408382) 6393896

[34] Kraus W, Glauner B, Höltje J-V 1985 UDP-*N*-acetylmuramylpentapeptide as acceptor in murein biosynthesis in *Escherichia coli* membranes and ether-permeabilized cells. J. Bacteriol. 162, 1000–1004. 3888951 10.1128/jb.162.3.1000-1004.1985 PMC215874

[35] Kraus W, Höltje J-V 1987 Two distinct transpeptidation reactions during murein synthesis in *Escherichia coli*. J. Bacteriol. 169, 3099–3103. 3298212 10.1128/jb.169.7.3099-3103.1987 PMC212355

[36] Metz R, Henning S, Hammes WP 1983 The complete sequence of murein synthesis in ether treated *Escherichia coli*. Arch. Microbiol. 136, 297–299. (10.1007/BF00425220) 6365012

[37] Perkins HR, Nieto M, Frere JM, Leyh-Bouille M, Ghuysen JM 1973 *Streptomyces* DD-carboxypeptidases as transpeptidases. The specificity for amino compounds acting as carboxyl acceptors. Biochem. J. 131, 707–718. 4722450 10.1042/bj1310707 PMC1177530

[38] Dusart J, Leyh-Bouille M, Ghuysen JM 1977 The peptidoglycan crosslinking enzyme system in *Streptomyces* strains R61, K15 and rimosus. Kinetic coefficients involved in the interactions of the membrane-bound transpeptidase with peptide substrates and beta-lactam antibiotics. Eur. J. Biochem. 81, 33–44. (10.1111/j.1432-1033.1977.tb11924.x) 590269

[39] Frere JM, Ghuysen JM, Perkins HR, Nieto M 1973 Kinetics of concomitant transfer and hydrolysis reactions catalysed by the exocellular DD-carboxypeptidase-transpeptidase of streptomyces R61. Biochem. J. 135, 483–492. 4772274 10.1042/bj1350483 PMC1165850

[40] Ghuysen JM, Leyh-Bouille M, Campbell JN, Moreno R, Frere JM, Duez C, Nieto M, Perkins HR 1973 Structure of the wall peptidoglycan of *Streptomyces* R39 and the specificity profile of its exocellular DD-carboxypeptidase-transpeptidase for peptide acceptors. Biochemistry 12, 1243–1251. (10.1021/bi00731a001) 4696752

[41] Anderson JS, Matsuhashi M, Haskin MA, Strominger JL 1967 Biosynthesis of the peptidoglycan of bacterial cell walls. II. Phospholipid carriers in the reaction sequence. J. Biol. Chem. 242, 3180–3190. 6027793

[42] Higashi Y, Strominger JL, Sweeley CC 1967 Structure of a lipid intermediate in cell wall peptidoglycan synthesis: a derivative of a C55 isoprenoid alcohol. Proc. Natl Acad. Sci. USA 57, 1878–1884. (10.1073/pnas.57.6.1878) 5231417 PMC224560

[43] Nakagawa J, Tamaki S, Matsuhashi M 1979 Purified penicillin-binding proteins 1B from *Escherichia coli* membrane showing activities of both peptidoglycan polymerase and peptidoglycan crosslinking enzyme. Agric. Biol. Chem. 43, 1379–1380. (10.1271/bbb1961.43.1379)

[44] Ishino F, Mitsui K, Tamaki S, Matsuhashi M 1980 Dual enzyme activities of cell wall peptidoglycan synthesis, peptidoglycan transglycosylase and penicillin-sensitive transpeptidase, in purified preparations of *Escherichia coli* penicillin-binding protein 1A. Biochem. Biophys. Res. Commun. 97, 287–293. (10.1016/S0006-291X(80)80166-5) 7006606

[45] Nakagawa J, Matsuhashi M 1982 Molecular divergence of a major peptidoglycan synthetase with transglycosylase–transpeptidase activities in *Escherichia coli*-penicillin-binding protein 1Bs. Biochem. Biophys. Res. Commun. 105, 1546–1553. (10.1016/0006-291X(82)90964-0) 7049165

[46] Tomioka S, Ishino F, Tamaki S, Matsuhashi M 1982 Formation of hyper-crosslinked peptidoglycan with multiple crosslinkages by a penicillin-binding protein, 1A, of *Escherichia coli*. Biochem. Biophys. Res. Commun. 106, 1175–1182. (10.1016/0006-291X(82)91236-0) 7052086

[47] Ishino F, Matsuhashi M 1981 Peptidoglycan synthetic enzyme activities of highly purified penicillin-binding protein 3 in *Escherichia coli*: a septum-forming reaction sequence. Biochem. Biophys. Res. Commun. 101, 905–911. (10.1016/0006-291X(81)91835-0) 7030331

[48] Bertsche U et al. 2006 Interaction between two murein (peptidoglycan) synthases, PBP3 and PBP1B, in *Escherichia coli*. Mol. Microbiol. 61, 675–690. (10.1111/j.1365-2958.2006.05280.x) 16803586

[49] Tamura T, Suzuki H, Nishimura Y, Mizoguchi J, Hirota Y 1980 On the process of cellular division in *Escherichia coli*: isolation and characterization of penicillin-binding proteins 1a, 1b, and 3. Proc. Natl Acad. Sci. USA 77, 4499–4503. (10.1073/pnas.77.8.4499) 7001458 PMC349871

[50] Jackson GE, Strominger JL 1984 Synthesis of peptidoglycan by high molecular weight penicillin-binding proteins of *Bacillus subtilis* and *Bacillus stearothermophilus*. J. Biol. Chem. 259, 1483–1490. 6420410

[51] Taku A, Stuckey M, Fan DP 1982 Purification of the peptidoglycan transglycosylase of *Bacillus megaterium*. J. Biol. Chem. 257, 5018–5022. 6802846

[52] Branstrom AA, Midha S, Goldman RC 2000 *In situ* assay for identifying inhibitors of bacterial transglycosylase. FEMS Microbiol. Lett. 191, 187–190. (10.1111/j.1574-6968.2000.tb09338.x) 11024262

[53] Vollmer W, Höltje J-V 2000 A simple screen for murein transglycosylase inhibitors. Antimicrob. Agents Chemother. 44, 1181–1185. (10.1128/AAC.44.5.1181-1185.2000) 10770749 PMC89842

[54] Schwartz B, Markwalder JA, Wang Y 2001 Lipid II: total synthesis of the bacterial cell wall precursor and utilization as a substrate for glycosyltransfer and transpeptidation by penicillin binding protein (PBP) 1b of *Escherichia coli*. J. Am. Chem. Soc. 123, 11 638–11 643. (10.1021/ja0166848) 11716719

[55] VanNieuwenhze MS, Mauldin SC, Zia-Ebrahimi M, Winger BE, Hornback WJ, Saha SL, Aikins JA, Blaszczak LC 2002 The first total synthesis of lipid II: the final monomeric intermediate in bacterial cell wall biosynthesis. J. Am. Chem. Soc. 124, 3656–3660. (10.1021/ja017386d) 11929255

[56] Ye XY, Lo MC, Brunner L, Walker D, Kahne D, Walker S 2001 Better substrates for bacterial transglycosylases. J. Am. Chem. Soc. 123, 3155–3156. (10.1021/ja010028q) 11457035

[57] Umbreit JN, Strominger JL 1972 Isolation of the lipid intermediate in peptidoglycan biosynthesis from *Escherichia coli*. J. Bacteriol. 112, 1306–1309. 4565540 10.1128/jb.112.3.1306-1309.1972 PMC251564

[58] Anderson JS, Matsuhashi M, Haskin MA, Strominger JL 1965 Lipid-phosphoacetylmuramyl-pentapeptide and lipid-phosphodisaccharide-pentapeptide: presumed membrane transport intermediates in cell wall synthesis. Proc. Natl Acad. Sci. USA 53, 881–889. (10.1073/pnas.53.4.881) 14324547 PMC221083

[59] Brötz H, Bierbaum G, Leopold K, Reynolds PE, Sahl HG 1998 The antibiotic mersacidin inhibits peptidoglycan synthesis by targeting lipid II. Antimicrob. Agents Chemother. 42, 154–160. 9449277 10.1128/aac.42.1.154 PMC105472

[60] Kramer NE, Smid EJ, Kok J, de Kruijff B, Kuipers OP, Breukink E 2004 Resistance of Gram-positive bacteria to nisin is not determined by lipid II levels. FEMS Microbiol. Lett. 239, 157–161. (10.1016/j.femsle.2004.08.033) 15451114

[61] van Heijenoort J 2001 Recent advances in the formation of the bacterial peptidoglycan monomer unit. Nat. Prod. Rep. 18, 503–519. (10.1039/a804532a) 11699883

[62] Watkinson RJ, Hussey H, Baddiley J 1971 Shared lipid phosphate carrier in the biosynthesis of teichoic acid and peptidoglycan. Nat. New Biol. 229, 57–59. (10.1038/newbio229057a0) 4250444

[63] Breukink E, van Heusden HE, Vollmerhaus PJ, Swiezewska E, Brunner L, Walker S, Heck AJ, de Kruijff B 2003 Lipid II is an intrinsic component of the pore induced by nisin in bacterial membranes. J. Biol. Chem. 278, 19 898–19 903. (10.1074/jbc.M301463200) 12663672

[64] Swiezewska E, Sasak W, Mańkowski T, Jankowski W, Vogtman T, Krajewska I, Hertel J, Skoczylas E, Chojnacki T 1994 The search for plant polyprenols. Acta Biochim. Pol. 41, 221–260. 7856395

[65] Hartley MD, Schneggenburger PE, Imperiali B 2013 Lipid bilayer nanodisc platform for investigating polyprenol-dependent enzyme interactions and activities. Proc. Natl Acad. Sci. USA 110, 20 863–20 870. (10.1073/pnas.1320852110) PMC3876266 24302767

[66] Swiezewska E, Chojnacki T 1991 Long-chain polyprenols from *Potentilla aurea*. Phytochemistry 30, 267–270. (10.1016/0031-9422(91)84135-F)

[67] Danilov LL, Druzhinina TN, Kalinchuk NA, Maltsev SD, Shibaev VN 1989 Polyprenyl phosphates: synthesis and structure–activity relationship for a biosynthetic system of *Salmonella anatum* O-specific polysaccharide. Chem. Phys. Lipids 51, 191–203. (10.1016/0009-3084(89)90006-6) 2611960

[68] Kohlrausch U, Höltje J-V 1991 One-step purification procedure for UDP-*N*-acetylmuramyl-peptide murein precursors from *Bacillus cereus*. FEMS Microbiol. Lett. 62, 253–257. (10.1111/j.1574-6968.1991.tb04451.x) 1904044

[69] Zawadzke LE, Wu P, Cook L, Fan L, Casperson M, Kishnani M, Calambur D, Hofstead SJ, Padmanabha R 2003 Targeting the MraY and MurG bacterial enzymes for antimicrobial therapeutic intervention. Anal. Biochem. 314, 243–252. (10.1016/S0003-2697(02)00622-X) 12654311

[70] Auger G, van Heijenoort J, Mengin-Lecreulx D, Blanot D 2003 A MurG assay which utilises a synthetic analogue of lipid I. FEMS Microbiol. Lett. 219, 115–119. (10.1016/S0378-1097(02)01203-X) 12594032

[71] van Dam V, Sijbrandi R, Kol M, Swiezewska E, de Kruijff B, Breukink E 2007 Transmembrane transport of peptidoglycan precursors across model and bacterial membranes. Mol. Microbiol. 64, 1105–1114. (10.1111/j.1365-2958.2007.05722.x) 17501931

[72] Mohammadi T, Sijbrandi R, Lutters M, Verheul J, Martin NI, den Blaauwen T, de Kruijff B, Breukink E 2014 Specificity of the transport of lipid II by FtsW in *Escherichia coli*. J. Biol. Chem. 289, 14 707–14 718. (10.1074/jbc.M114.557371) PMC4031526 24711460

[73] Figueiredo TA, Sobral RG, Ludovice AM, Almeida JM, Bui NK, Vollmer W, de Lencastre H, Tomasz A 2012 Identification of genetic determinants and enzymes involved with the amidation of glutamic acid residues in the peptidoglycan of *Staphylococcus aureus*. PLoS Pathogens 8, e1002508 (10.1371/journal.ppat.1002508) 22303291 PMC3267633

[74] Münch D, Roemer T, Lee SH, Engeser M, Sahl HG, Schneider T 2012 Identification and *in vitro* analysis of the GatD/MurT enzyme-complex catalyzing lipid II amidation in *Staphylococcus aureus*. PLoS Pathogens 8, e1002509 (10.1371/journal.ppat.1002509) 22291598 PMC3266927

[75] Zapun A, Philippe J, Abrahams KA, Signor L, Roper DI, Breukink E, Vernet T 2013 *In vitro* reconstitution of peptidoglycan assembly from the Gram-positive pathogen *Streptococcus pneumoniae*. ACS Chem. Biol. 8, 2688–2696. (10.1021/cb400575t) 24044435

[76] Hegde SS, Shrader TE 2001 FemABX family members are novel nonribosomal peptidyltransferases and important pathogen-specific drug targets. J. Biol. Chem. 276, 6998–7003. (10.1074/jbc.M008591200) 11083873

[77] Schneider T, Senn MM, Berger-Bächi B, Tossi A, Sahl HG, Wiedemann I 2004 *In vitro* assembly of a complete, pentaglycine interpeptide bridge containing cell wall precursor (lipid II-Gly5) of *Staphylococcus aureus*. Mol. Microbiol. 53, 675–685. (10.1111/j.1365-2958.2004.04149.x) 15228543

[78] Fonvielle M, Chemama M, Villet R, Lecerf M, Bouhss A, Valery JM, Etheve-Quelquejeu M, Arthur M 2009 Aminoacyl-tRNA recognition by the FemXWv transferase for bacterial cell wall synthesis. Nucleic Acids Res. 37, 1589–1601. (10.1093/nar/gkn1039) 19151092 PMC2655667

[79] Lovering AL, de Castro LH, Lim D, Strynadka NC 2007 Structural insight into the transglycosylation step of bacterial cell-wall biosynthesis. Science 315, 1402–1405. (10.1126/science.1136611) 17347437

[80] Barrett D, Wang TS, Yuan Y, Zhang Y, Kahne D, Walker S 2007 Analysis of glycan polymers produced by peptidoglycan glycosyltransferases. J. Biol. Chem. 282, 31 964–31 971. (10.1074/jbc.M705440200) PMC4048933 17704540

[81] Fraipont C et al. 2006 Glycosyl transferase activity of the *Escherichia coli* penicillin-binding protein 1b: specificity profile for the substrate. Biochemistry 45, 4007–4013. (10.1021/bi051055m) 16548528

[82] Perlstein DL, Wang TS, Doud EH, Kahne D, Walker S 2010 The role of the substrate lipid in processive glycan polymerization by the peptidoglycan glycosyltransferases. J. Am. Chem. Soc. 132, 48–49. (10.1021/ja909325m) 20017480 PMC2830065

[83] Perlstein DL, Zhang Y, Wang TS, Kahne DE, Walker S 2007 The direction of glycan chain elongation by peptidoglycan glycosyltransferases. J. Am. Chem. Soc. 129, 12 674–12 675. (10.1021/ja075965y) 17914829 PMC3206585

[84] Lovering AL, Gretes M, Strynadka NC 2008 Structural details of the glycosyltransferase step of peptidoglycan assembly. Curr. Opin Struct. Biol. 18, 534–543. (10.1016/j.sbi.2008.07.002) 18721881

[85] Zhang Y, Fechter EJ, Wang TS, Barrett D, Walker S, Kahne DE 2007 Synthesis of heptaprenyl-lipid IV to analyze peptidoglycan glycosyltransferases. J. Am. Chem. Soc. 129, 3080–3081. (10.1021/ja069060g) 17323951 PMC3222299

[86] Bury D et al. 2015 Positive cooperativity between acceptor and donor sites of the peptidoglycan glycosyltransferase. Biochem. Pharmacol. 93, 141–150. (10.1016/j.bcp.2014.11.003) 25462814

[87] Rebets Y, Lupoli T, Qiao Y, Schirner K, Villet R, Hooper D, Kahne D, Walker S 2014 Moenomycin resistance mutations in *Staphylococcus aureus* reduce peptidoglycan chain length and cause aberrant cell division. ACS Chem. Biol. 9, 459–467. (10.1021/cb4006744) 24255971 PMC3944067

[88] Barrett D, Leimkuhler C, Chen L, Walker D, Kahne D, Walker S 2005 Kinetic characterization of the glycosyltransferase module of *Staphylococcus aureus* PBP2. J. Bacteriol. 187, 2215–2217. (10.1128/JB.187.6.2215-2217.2005) 15743972 PMC1064046

[89] Reed P, Veiga H, Jorge AM, Terrak M, Pinho MG 2011 Monofunctional transglycosylases are not essential for *Staphylococcus aureus* cell wall synthesis. J. Bacteriol. 193, 2549–2556. (10.1128/JB.01474-10) 21441517 PMC3133172

[90] Terrak M, Nguyen-Disteche M 2006 Kinetic characterization of the monofunctional glycosyltransferase from *Staphylococcus aureus*. J. Bacteriol. 188, 2528–2532. (10.1128/JB.188.7.2528-2532.2006) 16547040 PMC1428434

[91] Terrak M, Ghosh TK, van Heijenoort J, Van Beeumen J, Lampilas M, Aszodi J, Ayala JA, Ghuysen JM, Nguyen-Disteche M 1999 The catalytic, glycosyl transferase and acyl transferase modules of the cell wall peptidoglycan-polymerizing penicillin-binding protein 1b of *Escherichia coli*. Mol. Microbiol. 34, 350–364. (10.1046/j.1365-2958.1999.01612.x) 10564478

[92] Zawadzka-Skomial J, Markiewicz Z, Nguyen-Disteche M, Devreese B, Frere JM, Terrak M 2006 Characterization of the bifunctional glycosyltransferase/acyltransferase penicillin-binding protein 4 of *Listeria monocytogenes*. J. Bacteriol. 188, 1875–1881. (10.1128/JB.188.5.1875-1881.2006) 16484198 PMC1426562

[93] Schwartz B, Markwalder JA, Seitz SP, Wang Y, Stein RL 2002 A kinetic characterization of the glycosyltransferase activity of *Escherichia coli* PBP1b and development of a continuous fluorescence assay. Biochemistry 41, 12 552–12 561. (10.1021/bi026205x) 12369847

[94] Offant J, Terrak M, Derouaux A, Breukink E, Nguyen-Disteche M, Zapun A, Vernet T 2010 Optimization of conditions for the glycosyltransferase activity of penicillin-binding protein 1a from *Thermotoga maritima*. FEBS J. 277, 4290–4298. (10.1111/j.1742-4658.2010.07817.x) 20849416

[95] Helassa N, Vollmer W, Breukink E, Vernet T, Zapun A 2012 The membrane anchor of penicillin-binding protein PBP2a from *Streptococcus pneumoniae* influences peptidoglycan chain length. FEBS J. 279, 2071–2081. (10.1111/j.1742-4658.2012.08592.x) 22487093

[96] Adam M et al. 1991 Acyltransferase activities of the high-molecular-mass essential penicillin-binding proteins. Biochem. J. 279, 601–604. 1953655 10.1042/bj2790601 PMC1151646

[97] Lebar MD et al. 2014 Reconstitution of peptidoglycan cross-linking leads to improved fluorescent probes of cell wall synthesis. J. Am. Chem. Soc. 136, 10 874–10 877. (10.1021/ja505668f) PMC4132960 25036369

[98] Lupoli TJ, Lebar MD, Markovski M, Bernhardt T, Kahne D, Walker S 2014 Lipoprotein activators stimulate *Escherichia coli* penicillin-binding proteins by different mechanisms. J. Am. Chem. Soc. 136, 52–55. (10.1021/ja410813j) 24341982 PMC3961711

[99] Qiao Y, Lebar MD, Schirner K, Schaefer K, Tsukamoto H, Kahne D, Walker S 2014 Detection of lipid-linked peptidoglycan precursors by exploiting an unexpected transpeptidase reaction. J. Am. Chem. Soc. 136, 14 678–14 681. (10.1021/ja508147s) PMC4210121 25291014

[100] Cava F, Kuru E, Brun YV, de Pedro MA 2013 Modes of cell wall growth differentiation in rod-shaped bacteria. Curr. Opin. Microbiol. 16, 731–737. (10.1016/j.mib.2013.09.004) 24094807 PMC3931007

[101] Kuru E, Hughes HV, Brown PJ, Hall E, Tekkam S, Cava F, de Pedro MA, Brun YV, VanNieuwenhze MS 2012 *In situ* probing of newly synthesized peptidoglycan in live bacteria with fluorescent D-amino acids. Angew. Chem. Int. Ed. Engl. 51, 12519–12523. (10.1002/anie.201206749) 23055266 PMC3589519

[102] Kuru E, Tekkam S, Hall E, Brun YV, Van Nieuwenhze MS 2015 Synthesis of fluorescent D-amino acids and their use for probing peptidoglycan synthesis and bacterial growth *in situ*. Nat. Protoc. 10, 33–52. (10.1038/nprot.2014.197) 25474031 PMC4300143

[103] Bertsche U, Breukink E, Kast T, Vollmer W 2005 *In vitro* murein peptidoglycan synthesis by dimers of the bifunctional transglycosylase-transpeptidase PBP1B from *Escherichia coli*. J. Biol. Chem. 280, 38 096–38 101. (10.1074/jbc.M508646200) 16154998

[104] Born P, Breukink E, Vollmer W 2006 *In vitro* synthesis of cross-linked murein and its attachment to sacculi by PBP1A from *Escherichia coli*. J. Biol. Chem. 281, 26 985–26 993. (10.1074/jbc.M604083200) 16840781

[105] Sung MT, Lai YT, Huang CY, Chou LY, Shih HW, Cheng WC, Wong CH, Ma C 2009 Crystal structure of the membrane-bound bifunctional transglycosylase PBP1b from *Escherichia coli*. Proc. Natl Acad. Sci. USA 106, 8824–8829. (10.1073/pnas.0904030106) 19458048 PMC2689995

[106] Typas A et al. 2010 Regulation of peptidoglycan synthesis by outer-membrane proteins. Cell 143, 1097–1109. (10.1016/j.cell.2010.11.038) 21183073 PMC3060616

[107] Glauner B 1988 Separation and quantification of muropeptides with high-performance liquid chromatography. Anal. Biochem. 172, 451–464. (10.1016/0003-2697(88)90468-X) 3056100

[108] Potluri L et al. 2010 Septal and lateral wall localization of PBP5, the major D,D-carboxypeptidase of *Escherichia coli*, requires substrate recognition and membrane attachment. Mol. Microbiol. 77, 300–323. (10.1111/j.1365-2958.2010.07205.x) 20545860 PMC2909392

[109] Spratt BG 1975 Distinct penicillin binding proteins involved in the division, elongation, and shape of *Escherichia coli* K12. Proc. Natl Acad. Sci. USA 72, 2999–3003. (10.1073/pnas.72.8.2999) 1103132 PMC432906

[110] Macheboeuf P, Contreras-Martel C, Job V, Dideberg O, Dessen A 2006 Penicillin binding proteins: key players in bacterial cell cycle and drug resistance processes. FEMS Microbiol. Rev. 30, 673–691. (10.1111/j.1574-6976.2006.00024.x) 16911039

[111] Marrec-Fairley M, Piette A, Gallet X, Brasseur R, Hara H, Fraipont C, Ghuysen JM, Nguyen-Disteche M 2000 Differential functionalities of amphiphilic peptide segments of the cell-septation penicillin-binding protein 3 of *Escherichia coli*. Mol. Microbiol. 37, 1019–1031. (10.1046/j.1365-2958.2000.02054.x) 10972821

[112] Sauvage E et al. 2014 Crystal structure of penicillin-binding protein 3 (PBP3) from *Escherichia coli*. PLoS ONE 9, e98042 (10.1371/journal.pone.0098042) 24875494 PMC4038516

[113] Banzhaf M et al. 2012 Cooperativity of peptidoglycan synthases active in bacterial cell elongation. Mol. Microbiol. 85, 179–194. (10.1111/j.1365-2958.2012.08103.x) 22606933

[114] Burman LG, Park JT 1984 Molecular model for elongation of the murein sacculus of *Escherichia coli*. Proc. Natl Acad. Sci. USA 81, 1844–1848. (10.1073/pnas.81.6.1844) 6369331 PMC345018

[115] de Jonge BL, Wientjes FB, Jurida I, Driehuis F, Wouters JT, Nanninga N 1989 Peptidoglycan synthesis during the cell cycle of *Escherichia coli*: composition and mode of insertion. J. Bacteriol. 171, 5783–5794. 2681142 10.1128/jb.171.11.5783-5794.1989 PMC210437

[116] Nguyen-Disteche M, Fraipont C, Buddelmeijer N, Nanninga N 1998 The structure and function of *Escherichia coli* penicillin-binding protein 3. Cell Mol. Life Sci. 54, 309–316. (10.1007/s000180050157) 9614966 PMC11147360

[117] Bernard E, Rolain T, Courtin P, Hols P, Chapot-Chartier MP 2011 Identification of the amidotransferase AsnB1 as being responsible for meso-diaminopimelic acid amidation in *Lactobacillus plantarum* peptidoglycan. J. Bacteriol. 193, 6323–6330. (10.1128/JB.05060-11) 21949063 PMC3209238

[118] Levefaudes M, Patin D, de Sousa-d'Auria C, Chami M, Blanot D, Herve M, Arthur M, Houssin C, Mengin-Lecreulx D 2015 Diaminopimelic acid amidation in Corynebacteriales: new insights into the role of LtsA in peptidoglycan modification. J. Biol. Chem. 290, 13 079–13 094. (10.1074/jbc.M115.642843) 25847251 PMC4505564

[119] Mainardi JL, Villet R, Bugg TD, Mayer C, Arthur M 2008 Evolution of peptidoglycan biosynthesis under the selective pressure of antibiotics in Gram-positive bacteria. FEMS Microbiol. Rev. 32, 386–408. (10.1111/j.1574-6976.2007.00097.x) 18266857

[120] Mohammadi T et al. 2011 Identification of FtsW as a transporter of lipid-linked cell wall precursors across the membrane. EMBO J. 30, 1425–1432. (10.1038/emboj.2011.61) 21386816 PMC3102273

[121] Sham LT, Butler EK, Lebar MD, Kahne D, Bernhardt TG, Ruiz N 2014 MurJ is the flippase of lipid-linked precursors for peptidoglycan biogenesis. Science 345, 220–222. (10.1126/science.1254522) 25013077 PMC4163187

[122] Paradis-Bleau C, Markovski M, Uehara T, Lupoli TJ, Walker S, Kahne DE, Bernhardt TG 2010 Lipoprotein cofactors located in the outer membrane activate bacterial cell wall polymersases. Cell 143, 1110–1120. (10.1016/j.cell.2010.11.037) 21183074 PMC3085243

[123] Egan AJF et al. 2014 Outer-membrane lipoprotein LpoB spans the periplasm to stimulate the peptidoglycan synthase PBP1B. Proc. Natl Acad. Sci. USA 111, 8197–8202. (10.1073/pnas.1400376111) 24821816 PMC4050580

[124] Müller P et al. 2007 The essential cell division protein FtsN interacts with the murein (peptidoglycan) synthase PBP1B in *Escherichia coli*. J. Biol. Chem. 282, 36 394–36 402. (10.1074/jbc.M706390200) 17938168

[125] Gray AN et al. 2015 Coordination of peptidoglycan synthesis and outer membrane constriction during *Escherichia coli* cell division. eLife 4, e07118 (10.7554/eLife.07118) PMC4458516 25951518

[126] Dorr T, Lam H, Alvarez L, Cava F, Davis BM, Waldor MK 2014 A novel peptidoglycan binding protein crucial for PBP1A-mediated cell wall biogenesis in *Vibrio cholerae*. PLoS Genet. 10, e1004433 (10.1371/journal.pgen.1004433) 24945690 PMC4063736

[127] Mercer KL, Weiss DS 2002 The *Escherichia coli* cell division protein FtsW is required to recruit its cognate transpeptidase, FtsI (PBP3), to the division site. J. Bacteriol. 184, 904–912. (10.1128/jb.184.4.904-912.2002) 11807049 PMC134820

[128] Claessen D, Emmins R, Hamoen LW, Daniel RA, Errington J, Edwards DH 2008 Control of the cell elongation–division cycle by shuttling of PBP1 protein in *Bacillus subtilis*. Mol. Microbiol. 68, 1029–1046. (10.1111/j.1365-2958.2008.06210.x) 18363795

[129] Hocking J, Priyadarshini R, Takacs CN, Costa T, Dye NA, Shapiro L, Vollmer W, Jacobs-Wagner C 2012 Osmolality-dependent relocation of penicillin-binding protein PBP2 to the division site in *Caulobacter crescentus*. J. Bacteriol. 194, 3116–3127. (10.1128/JB.00260-12) 22505677 PMC3370875

[130] Yousif SY, Broome-Smith JK, Spratt BG 1985 Lysis of *Escherichia coli* by beta-lactam antibiotics: deletion analysis of the role of penicillin-binding proteins 1A and 1B. J. Gen. Microbiol. 131, 2839–2845. 3906031 10.1099/00221287-131-10-2839

[131] King DT, Lameignere E, Strynadka NC 2014 Structural insights into the lipoprotein outer membrane regulator of penicillin-binding protein 1B. J. Biol. Chem. 289, 19 245–19 253. (10.1074/jbc.M114.565879) PMC4081958 24808177

[132] Jean NL et al. 2014 Elongated structure of the outer-membrane activator of peptidoglycan synthesis LpoA: implications for PBP1A stimulation. Structure 22, 1047–1054. (10.1016/j.str.2014.04.017) 24954617 PMC4111904

[133] Krachler AM, Sharma A, Kleanthous C 2010 Self-association of TPR domains: Lessons learned from a designed, consensus-based TPR oligomer. Proteins 78, 2131–2143. (10.1002/prot.22726) 20455268

[134] Das AK, Cohen PTW, Barford D 1998 The structure of the tetratricopeptide repeats of protein phosphatase 5: implications for TPR-mediated protein–protein interactions. EMBO J. 17, 1192–1199. (10.1093/emboj/17.5.1192) 9482716 PMC1170467

[135] Vijayalakshmi J, Akerley BJ, Saper MA 2008 Structure of YraM, a protein essential for growth of *Haemophilus influenzae*. Proteins 73, 204–217. (10.1002/prot.22033) 18412262

[136] Aarsman ME, Piette A, Fraipont C, Vinkenvleugel TM, Nguyen-Disteche M, den Blaauwen T 2005 Maturation of the *Escherichia coli* divisome occurs in two steps. Mol. Microbiol. 55, 1631–1645. (10.1111/j.1365-2958.2005.04502.x) 15752189

[137] Egan AJF, Vollmer W 2013 The physiology of bacterial cell division. Ann. N.Y. Acad. Sci. 1277, 8–28. (10.1111/j.1749-6632.2012.06818.x) 23215820

[138] Ursinus A, van den Ent F, Brechtel S, de Pedro M, Höltje J-V, Löwe J, Vollmer W 2004 Murein (peptidoglycan) binding property of the essential cell division protein FtsN from *Escherichia coli*. J. Bacteriol. 186, 6728–6737. (10.1128/JB.186.20.6728-6737.2004) 15466024 PMC522186

[139] Yang JC, Van Den Ent F, Neuhaus D, Brevier J, Löwe J 2004 Solution structure and domain architecture of the divisome protein FtsN. Mol. Microbiol. 52, 651–660. (10.1111/j.1365-2958.2004.03991.x) 15101973

[140] Liu B, Persons L, Lee L, de Boer PA 2015 Roles for both FtsA and the FtsBLQ subcomplex in FtsN-stimulated cell constriction in *Escherichia coli*. Mol. Microbiol. 95, 945–970. (10.1111/mmi.12906) 25496160 PMC4428282

[141] Derouiche R, Benedetti H, Lazzaroni JC, Lazdunski C, Lloubes R 1995 Protein complex within *Escherichia coli* inner membrane. TolA N-terminal domain interacts with TolQ and TolR proteins. J. Biol. Chem. 270, 11 078–11 084. (10.1074/jbc.270.19.11078) 7744737

[142] Cascales E, Gavioli M, Sturgis JN, Lloubes R 2000 Proton motive force drives the interaction of the inner membrane TolA and outer membrane pal proteins in *Escherichia coli*. Mol. Microbiol. 38, 904–915. (10.1046/j.1365-2958.2000.02190.x) 11115123

[143] Bonsor DA et al. 2009 Allosteric beta-propeller signalling in TolB and its manipulation by translocating colicins. EMBO J. 28, 2846–2857. (10.1038/emboj.2009.224) 19696740 PMC2750012

[144] Gerding MA, Ogata Y, Pecora ND, Niki H, de Boer PA 2007 The *trans*-envelope Tol–Pal complex is part of the cell division machinery and required for proper outer-membrane invagination during cell constriction in *E. coli*. Mol. Microbiol. 63, 1008–1025. (10.1111/j.1365-2958.2006.05571.x) 17233825 PMC4428343

[145] Krachler AM, Sharma A, Cauldwell A, Papadakos G, Kleanthous C 2010 TolA modulates the oligomeric status of YbgF in the bacterial periplasm. J. Mol. Biol. 403, 270–285. (10.1016/j.jmb.2010.08.050) 20816983

[146] Goley ED, Yeh YC, Hong SH, Fero MJ, Abeliuk E, McAdams HH, Shapiro L 2011 Assembly of the *Caulobacter* cell division machine. Mol. Microbiol. 80, 1680–1698. (10.1111/j.1365-2958.2011.07677.x) 21542856 PMC3707389

[147] Cleverley RM et al. 2014 Structure and function of a spectrin-like regulator of bacterial cytokinesis. Nat. Commun. 5, 5421 (10.1038/ncomms6421) 25403286 PMC4243239

[148] Levin PA, Kurtser IG, Grossman AD 1999 Identification and characterization of a negative regulator of FtsZ ring formation in *Bacillus subtilis*. Proc. Natl Acad. Sci. USA 96, 9642–9647. (10.1073/pnas.96.17.9642) 10449747 PMC22263

[149] Holloway PW 1973 A simple procedure for removal of Triton X-100 from protein samples. Anal. Biochem. 53, 304–308. (10.1016/0003-2697(73)90436-3) 4711106

[150] Hope MJ, Bally MB, Webb G, Cullis PR 1985 Production of large unilamellar vesicles by a rapid extrusion procedure: characterization of size distribution, trapped volume and ability to maintain a membrane potential. Biochim. Biophys. Acta 812, 55–65. (10.1016/0005-2736(85)90521-8) 23008845

[151] Levy D, Bluzat A, Seigneuret M, Rigaud JL 1990 A systematic study of liposome and proteoliposome reconstitution involving Bio-Bead-mediated Triton X-100 removal. Biochim. Biophys. Acta 1025, 179–190. (10.1016/0005-2736(90)90096-7) 2364077

[152] Biboy J, Bui NK, Vollmer W 2013 *In vitro* peptidoglycan synthesis assay with lipid II substrate. Methods Mol. Biol. 966, 273–288. (10.1007/978-1-62703-245-2_17) 23299741

